# Evaluating the Bias in Hospital Data: Automatic Preprocessing of Patient Pathways Algorithm Development and Validation Study

**DOI:** 10.2196/58978

**Published:** 2024-09-23

**Authors:** Laura Uhl, Vincent Augusto, Benjamin Dalmas, Youenn Alexandre, Paolo Bercelli, Fanny Jardinaud, Saber Aloui

**Affiliations:** 1 Mines Saint-Etienne Centre Ingénierie Santé Unité Mixte de Recherche (UMR) 6158 Laboratoire d'Informatique, de Modélisation et d'Optimisation des Systèmes (LIMOS) Centre national de la recherche scientifique (CNRS) Saint-Etienne France; 2 Groupe Hospitalier Bretagne Sud Lorient France; 3 Direction Anticipation & Usages Enovacom Marseille France; 4 Inserm, UMR 1085 Ester Centre Hospitalier Universitaire Angers Angers France

**Keywords:** preprocessing, framework, health care data, patient pathway, bed management

## Abstract

**Background:**

The optimization of patient care pathways is crucial for hospital managers in the context of a scarcity of medical resources. Assuming unlimited capacities, the pathway of a patient would only be governed by pure medical logic to meet at best the patient’s needs. However, logistical limitations (eg, resources such as inpatient beds) are often associated with delayed treatments and may ultimately affect patient pathways. This is especially true for unscheduled patients—when a patient in the emergency department needs to be admitted to another medical unit without disturbing the flow of planned hospitalizations.

**Objective:**

In this study, we proposed a new framework to automatically detect activities in patient pathways that may be unrelated to patients’ needs but rather induced by logistical limitations.

**Methods:**

The scientific contribution lies in a method that transforms a database of historical pathways with bias into 2 databases: a labeled pathway database where each activity is labeled as relevant (related to a patient’s needs) or irrelevant (induced by logistical limitations) and a corrected pathway database where each activity corresponds to the activity that would occur assuming unlimited resources. The labeling algorithm was assessed through medical expertise. In total, 2 case studies quantified the impact of our method of preprocessing health care data using process mining and discrete event simulation.

**Results:**

Focusing on unscheduled patient pathways, we collected data covering 12 months of activity at the Groupe Hospitalier Bretagne Sud in France. Our algorithm had 87% accuracy and demonstrated its usefulness for preprocessing traces and obtaining a clean database. The 2 case studies showed the importance of our preprocessing step before any analysis. The process graphs of the processed data had, on average, 40% (SD 10%) fewer variants than the raw data. The simulation revealed that 30% of the medical units had >1 bed difference in capacity between the processed and raw data.

**Conclusions:**

Patient pathway data reflect the actual activity of hospitals that is governed by medical requirements and logistical limitations. Before using these data, these limitations should be identified and corrected. We anticipate that our approach can be generalized to obtain unbiased analyses of patient pathways for other hospitals.

## Introduction

### Context

Bed management is a critical task for hospitals to provide coherent care pathways. Daily bed management consists of finding beds for patients coming from the emergency department (ED) in appropriate medical units without canceling planned hospitalizations. Therefore, bed management involves 2 distinct flows: unscheduled flow (life-threatening emergencies and patients coming to the ED) and scheduled flow (planned hospitalizations). Despite the complexity of the task, bed management is most often organized without the help of any decision support tools and involves multiple phone calls to find a bed in a medical unit matching the patient’s needs [1]. When medical units are facing high occupation rates, it is not always possible to find a bed to match patient needs.

In these situations, patients are either kept in the short-stay hospitalization unit of the ED or transferred to an overflow medical unit to wait for a bed. Consequently, the medical units visited by a patient do not always correspond to their medical needs. For example, a patient from the ED can be transferred to a surgery unit and then to a cardiology unit. This is the pathway observed in the data. The patient did not receive any surgical treatment. He was admitted to the surgery unit waiting for a cardiology unit bed. Therefore, the location of the patient does not always match the cause of hospitalization. The succession of medical units is called a patient pathway. Unscheduled pathways describe the pathways of patients coming from the ED. In this work, we only considered patients who visited the ED and were subsequently hospitalized.

The study of patient pathways reveals several challenges due to the variety of pathways, the lack of complete guidelines and references, and the heterogeneity of patient management between hospitals (due to equipment and organizational differences). Unscheduled pathways are difficult to explain because management rules or clear indicators are not available to identify them. In addition, the high number of pathway variants makes individual studies of each pathway impossible (eg, >1000 variants for French hospitals of average size) [2]. Process mining is an interesting tool for studying a set of pathways with several variants because a pathway can be seen as a patient care process [2,3]. Nevertheless, a large variance in pathways leads to uninterpretable process graphs. Strategies exist to make a process graph easier to read, such as trace clustering or graph size reduction using filters or aggregation [2], but these methods cannot identify which activities are relevant and which activities are induced by logistical limitations.

In this study, we sought to develop a method to assess observed pathways extracted from a hospital information system. We wanted to identify which medical units matched the cause of hospitalization (relevant) or not (irrelevant) in a patient pathway. The medical relevance or the relevance of treatments was not evaluated, nor was the choice of the bed manager. Only the relevance of the patient’s location was evaluated. An *irrelevant* medical unit means that the patient would have been hospitalized in another unit if there were an infinite number of beds. The identification of such irrelevance is important to avoid any misinterpretation of further analysis results. In this paper, we often use the word *bias* to denote a wrong, inaccurate, or incomplete interpretation of a real situation because the data do not represent reality. We use the expression *bias in pathways* or *data bias* to refer to data that represent pathways that do not always correspond to patients’ medical needs.

### Related Work

We did not find proper literature on the task of assessing pathways but, rather, heterogeneous papers dealing with bias or phases of a pathway. In 1989, Selker et al [4] designed the “Delay Tool,” which detects medically unnecessary hospital days. It is based on a taxonomy of delays. Each stay was manually evaluated using patient records with the Delay Tool method. In an article on the prediction of the disposition of ED patients, El-Bouri et al [5] considered the fact that ED patients can be admitted to an inpatient unit inappropriate for their diagnosis. Patients were filtered according to whether their primary diagnosis code for the ED visit clearly corresponded to the admission inpatient unit. Their aim was to avoid learning from biased data. These methods require a thesaurus of all possible diagnoses linked to appropriate wards. To study patient pathways, Franck et al [6] designed a generic framework to model pathways and distinguished 3 different phases: (1) a waiting phase—the patient waits in the ED (unscheduled) or at home (scheduled) to be admitted to the relevant medical unit, (2) an acute phase—the patient receives care in the medical unit, and (3) a rehabilitative phase—rehabilitative care of the patient. They also differentiated scheduled patients from unscheduled patients. To analyze the clinical pathways, they defined relevant pathways for each type of patient by considering only the acute phase and substitution options. To identify the relevant pathways and substitutions, they used process mining on administrative data. This method is very accurate but time-consuming given that a relevant pathway and substitutions must be defined for each pathology. They applied this method exclusively to patients with stroke.

Data quality in health research is a shared problem, and solutions have been proposed to improve several dimensions of quality [7]. However, methods are often not suggested to correct specific bias in health care data due to missing details about a piece of information.

Patient pathways can be seen as processes, with the succession of medical units being the succession of events. Therefore, pathways can be studied using process mining techniques. “The goal of process mining is to use event data to extract process-related information” [8]. The first rough representation of patient pathways using process discovery algorithms provides a spaghettilike process model. Indeed, process discovery algorithms are not successful with event logs that involve numerous variants and many events [2]. A typical solution to untangle a spaghettilike model is to cluster the whole set of traces (trace clustering) and represent each cluster using a process model that should be smaller and more comprehensive. The main challenges of the clustering of patient pathways are the integration of medical knowledge (medical logic) and the evaluation of the resulting clusters. In the literature, several methods for trace clustering have been proposed. Some of these methods are distance-based clustering algorithms. The core of these methods is to compute distances between traces to apply classic clustering algorithms (trace clustering [9], trace clustering based on conserved patterns [10], context-aware clustering [11], and the method by Delias et al [12]). Others are model based; these methods gradually build a process model that represents a cluster, and a trace is assigned to the cluster with the nearest process model (sequence clustering [13], active trace clustering [14], disjunctive workflow schema [15], graph-based approach and Markov models [16], and behavioral topic analysis [17]). In terms of cluster evaluation, different metrics are used. Some metrics analyze cluster intrahomogeneity, and others analyze the complexity of the process model of each cluster. There is no consensus on these metrics, and they do not guarantee that the clusters computed using the algorithm have an expert logic. Hence, trace clustering does not give us complete satisfaction in characterizing pathways. Another approach for simplifying process models was proposed by Fahland and Van der Aalst [18] based on unfolding.

Some data preparation techniques can also reduce the “spaghettiness” of process models. Data preparation is an unavoidable step in a process mining project and impacts on the resulting process graph, as highlighted by De Roock and Martin [19] in their most recent state-of-the-art study. Several methods have been suggested in the literature to simplify process models. Semantic log purging was proposed by Ly et al [20] in 2012 to clean log data. This method is based on the identification of “fundamental constraints that a process has to obey” thanks to experts. Only a qualitative evaluation and 1 experiment using 1 dataset were performed. Van Zelst et al [21] reviewed the literature on event abstraction in process mining. However, this technique is not related to the problem addressed in this study because our dataset did not provide information on the granularity of events. Several papers address the issue of time stamp inaccuracy.

Martin et al [22] proposed interactive data cleaning. Dixit et al [23] created a method to detect and repair event ordering mistakes. Rogge-Solti et al [24] presented a similar approach to repairing missing events based on alignment. In addition, these researchers created a method for time repairing.

To rigorously prepare the data and event log, different frameworks have been developed. Andrews et al [25] applied the Cross-Industry Standard Process for Data Mining method to identify data quality issues. The data quality dimensions used in data mining are also useful for assessing data quality in process mining. Nevertheless, the researchers do not consider dimensions specific to processes, such as trace coherence. Therefore, the fourth step, namely, prestudy process mining analysis, is important to assess this dimension. Bose et al [26] noted 27 event log quality issues based on 4 categories (missing data, incorrect data, imprecise data, and irrelevant data) and 9 components of an event log (case, event, belongs to, case attributes, event attributes, position, activity name, time stamp, and resource). The researchers also distinguished 4 process characteristics: (1) voluminous data, which refers to a large number of cases or events; (2) case heterogeneity, which refers to a large number of distinct traces; (3) event granularity, which refers to a large number of distinct activities; and (4) process flexibility and concept drifts. The issues caused by case heterogeneity are a part of the problem we attempted to address in this study. Van Eck et al [27] suggested PM^2^, a process mining project methodology. Data processing is the third step and consists of creating views (creating the event log), aggregating events, enriching logs (addition of attributes), and filtering logs. Vanbrabant et al [28] presented a data quality framework based on 3 previous frameworks and applied it to a case study—pretreatment of ED data before simulation. These researchers divided quality problems into hierarchical classes. Verhulst [29] defined very precisely the different data quality dimensions for process mining and their scoring methods. All these papers on data preparation are general to process mining datasets and do not answer the question of pathway bias.

Process mining is not the only method used to analyze patient pathways, and this method can be combined with other methods such as discrete event simulation (DES). Prodel et al [30,31] developed a framework to automatically convert a process model discovered using process mining into a simulation model of clinical pathways. Abohamad et al [32] used process mining to discover ED processes and then used DES to study bottlenecks. Wood and Murch [33] modeled patient pathways using Markov chains to study transfer delays between medical units and discharge delays. Karakra et al [34] also used a DES to model an ED and added a real-time connection to real-time patient data to create a digital twin. The digital twin of the patient enables the monitoring of their pathway and activities as well as near-future predictions. Some models reproduce an entire hospital. Holm et al [35] used a DES to model an entire hospital and patient flows through the wards and determine bed use. Demir et al [36] used a similar model to anticipate an increase in the number of patients and adapt resources. Ordu et al [37] achieved an even more complete model of a hospital and patient flows.

To conclude, this review of the literature reveals that process mining and simulation are the principal methods used to study patient pathways. Process mining is a standard tool for discovering patient pathways (or other health care processes), but important limitations are noted in the literature, especially the complexity of the model graphs. Data preparation techniques and clustering methods are suggested to compensate for this issue. Some methods are based on expert interviews or expert knowledge integration. This is similar to our construction of rules. Several papers focus on time stamp correction or missing events or labels, but none of the studies focus on our problem of biased events. Simulations do not consider input data quality. In this study, we focused on enriching the log by adding an attribute that can define an event as relevant or irrelevant.

### Objectives

The objective of this paper was twofold: (1) building a framework to model and analyze patient pathways and (2) proposing a method to automatically identify bias in patient pathway data. In other words, this method turns a database of observed pathways with bias into 2 databases: one database includes labeled pathways (identified bias), and one database includes corrected pathways (without bias). Such a method is intended to ease the preprocessing of real data for data analysts or hospital managers who seek a clean database with unbiased medical pathways.

The scientific contributions of this paper are as follows:

1. This study provides a new framework to model patient pathways considering hospital management constraints (eg, bed occupancy and resource availability). This framework is used to assess patient pathways.
2. This study develops a new method to automatically label and correct pathways based on hospital data. Pathway labeling aims to identify the steps in a patient pathway due to a difficult bed management, and path correction aims to correct irrelevant activities. The labeling algorithm was assessed by comparing its outputs with experts’ answers.
3. Two case studies are reported: (1) a quantitative comparison of the observed pathway database and the corrected pathway database was performed based on process mining using process model comparison and classical process mining indicators, and (2) a DES model based on the observed pathway database and the corrected pathway database was used to evaluate the impacts of data correction on the occupation of medical units.

## Methods

### Unscheduled Hospital Pathway Modeling Framework

#### Formal Definition of the Framework for the Study Patient Pathway

##### Overview

In this section, we propose a set of definitions that will be used to formalize the unscheduled hospital pathway modeling framework.

**Figure 1:**
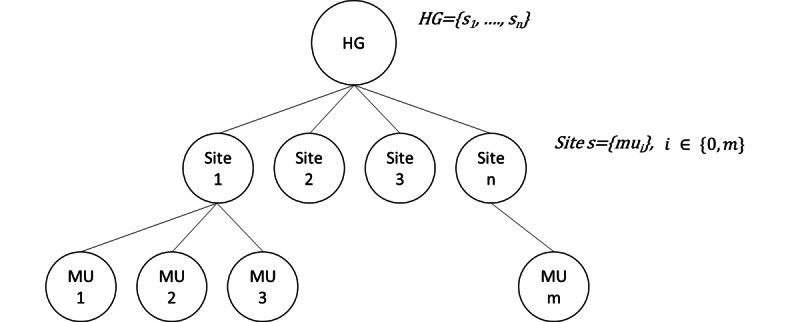
Hospital group (HG; {s1,..., sn}) structure. MU: medical unit.

In this study, we were interested in the medicine, surgery, obstetrics, and odontology (MCO). In French, the initials MCO stand for *Médecine, Chirurgie, Obstétrique et Odontologie* (medicine, surgery, obstetrics, and odontology). The medical units belong to a *hospital* that itself can belong to a *hospital group*. Figure 1 represents the dependencies among the hospital group, site, and medical unit. Here, we are interested in the pathways inside the same hospital group, which we call the *MCO-stay*. Multimedia Appendix 1 provides detailed definitions of the aforementioned concepts.

The hospital pathways are defined using a process mining formalism [8].

##### Definition 1 (Event)

Let *E* be the event universe (ie, the set of all possible event identifiers), E* be the set of all sequences over *E*, and *T* be the time domain. We assume that events are defined by several attributes; however, the case ID, time stamp, and activity name are mandatory for case identification, trace ordering, and event labeling, respectively.

Let *AN* be a set of attribute names. For any event *e* ∈ *E* and name z ∈ *AN*, *#_n_(e)* is the value of the attribute *z* for event *e*. We consider *#_activity_* ∈ *E* → *A* and #_time_ ∈ *E* → *T* functions that assign an activity name from a finite set of process activities *A* and a time stamp, respectively, to each event. For convenience, we assume the following standard attributes: (1) #*_activity_*(e) is the activity associated with event *e*, (2) #*_time_*(e) is the time stamp of event *e*, and (3) #*_trans_*(e) is the transaction type associated with event *e* (eg, schedule, start, complete, and suspend).

The transaction type attribute #*_trans_*(e) refers to the life cycle of activities. In most situations, activities take time. Therefore, events may point out, for example, the start or completion of activities.

##### Definition 2 (Trace)

A trace is a finite sequence of events denoted as *σ =< e_1,_ e_2_,..., e_n_ >* ∀*e_i_* ∈ *E*^∗^ such that each event appears only once: *e_i_* ≠*e_j_* for *1≤i<j≤|σ|*.

Specifically, *|σ|* denotes the length of the trace. In this case, *|σ| = n*.

##### Definition 3 (Stage)

In a trace, several events can have the same activity name. In the following, the subset of events with the same activity name that contains a single start event and a single completion event subsequent to the start event is referred to as a stage.

Let *e_i_* and *e_m_* such that (1) #*_activity_*(e_i_) = #*_activity_*(e_m_)=a; (2) #*_trans_*(e_i_)=start and #*_trans_*(e_m_)=complete; (3) #*_time_*(e_i_)=t_i_ ≤ #*_time_*(e_m_)=t_m_; and (4) ∄ e_j_ such that #*_time_*(e_j_)=t_j_≥t_i_, t_j_≤t_m_, #*_activity_*(e_j_)=a, and #*_trans_*(e_j_)=complete or #*_trans_*(e_j_)=start.

Stage = {e_j_|#*_activity_*(e_j_) = a and t_i_ ≤ t_j_ ≤ t_m_}

The duration of a stage is defined as the time between the start of the stage and its completion:

#*_duration_*(s) = #*_time_*(e_m_) – #*_time_*(e_i_)

In the following, we will note *e_x1,_ e_x2_,..., e_xi_* as all the events that compose stage *x*.

Furthermore, the duration of the trace σ of length *n* is determined as follows:

*duration*(σ) = #*_time_*(*e_n_*) – #*_time_*(*e*_1_)

In our study framework, a trace always begins at the ED stage and ends at the last unit of the MCO stay (the unit before the MCO discharge).

##### Definition 4 (Event Log)

An event log is a set of traces representing the execution of the underlying process. An event can only occur in 1 trace; however, events from different traces can share the same activity.

##### Definition 5 (Patient Pathway)

A patient pathway describes the succession of medical events inside a health care facility. In this work, each pathway is linked to an MCO stay.

A patient pathway is a set (*p, s, σ, d*) where *p* ∈ N is the identifier of the patient, *s* ∈ N is the identifier of the MCO stay, *σ* is the trace of the MCO stay, and *d* is the MCO discharge disposition.

We consider two types of pathways:

1. Scheduled pathways
  : these pathways are planned before patient admission.
2. Unscheduled pathways
  : neither the admissions nor the pathways are planned. The patients are hospitalized from the ED or admitted to a specific unit for life-threatening emergencies.

##### Definition 6 (Relevance of Stage)

A stage of a patient pathway is relevant if it is adequate that, at this moment, the patient is still hospitalized (first condition) and if the patient is in the medical unit intended to care for their pathology (second condition).

We consider 3 levels of relevance: level 2 (both conditions are met), level 1 (the second condition is not met; ie, the patient is not hospitalized in the ideal medical unit for their pathology), and level 0 (no condition is met, and there is no medical reason that justifies the patient being still hospitalized in this discipline).

##### Definition 7 (Bias)

In our context, a bias in the data is noted when some details about a piece of information are missing, which leads to a misinterpretation of a situation.

For example, a pathway {ED, Surgery, Geriatrics} without additional information suggests that the patient needs surgery after the ED followed by geriatric care. The bias is that the patient just stays in surgery while waiting for a bed in geriatrics.

##### Definition 8 (Activity Labels)

In this study, we consider 2 levels of activity names.

In level 1, *U* is the set of labels corresponding to all the names or IDs of the medical units constituting the hospital group. Consequently, an event activity is a medical unit that a patient has visited.

GH = {mu_1_,..., mu_n_}

U = {id(mu_i_)} with i ∈ [1, n]

#*_activity_*(e_x_) = id(mu_m_)

In level 2, let *L* be the set of labels corresponding to the relevance levels. *A* is the set of labels corresponding to the product of *U* and *L*:

L = {level 0, level 1, level 2}

A = U × L

#_activity_(e_x_) = (id(mu_m_), level l)

Hence, level 1 characterizes the activity of an event based on the ID of the medical unit, and level 2 adds a level of relevance. For more convenient reading, in the following sections, the activity of an event will be noted using the name of the medical unit.

#### Motivation

The pathway of a patient is governed not only by pure medical logic (health care needs) but also by logistical limitations. In other words, the pathway of a patient depends not only on their medical needs but also on the availability of inpatient beds and the possibility of discharge. Therefore, a patient can go to an unsuitable medical unit (unit *b*) because of a lack of beds in the suitable unit (unit *a*). The patient can later be transferred to unit *a*. Discharge also has an impact on patient pathways. Indeed, patients do not always immediately leave the hospital when they are medically fit for discharge because they are waiting for a discharge disposition. The challenge is to automatically identify these irrelevant steps in any MCO pathway. Indeed, these pathways are not clearly identified in the electronic health records (EHRs), and there is no generally applicable thesaurus of ideal pathways and no clear indicator of the adequacy of a unit in the EHR. The same medical unit can have different functions (see Textbox 1 for an example), and identical patients in terms of pathology can have different pathways according to hospital occupancy [6].

Our objective was to find a function (an algorithm) that evaluates the relevance of each stage. It is important to understand the word *relevance* as defined in the previous sections (*Definition 6: Relevance of Stage* section). Medical practices or medications were not judged here. Only the relevance of the patient’s location was evaluated. In our framework, an input trace with event labels comprising only the activity name is converted into an output trace with event labels comprising the activity name and the level of relevance.

**Example of the different roles that a medical unit can play in a patient pathway.**

**Examples**

- Emergency department (ED) to neurology: acute care in neurology
- ED to neurology to neurovascular intensive care: waiting in neurology for a bed in neurovascular intensive care
- ED to neurovascular intensive care to neurology: waiting for a discharge solution in neurology

#### Definition of the Function Evaluating the Relevance of Stages

Let *σ =< e_11_, e_12_, e_nx_ >* ∀*e_xi_*, #_activity_(e_xi_) ∈ U, and *σ’ =< e’_11_, e’_12_,..., e’_nx_ >* ∀*e’_xi_*, #*_activity_*(e’_xi_) ∈ *A*.

σ and σ*^′^* are 2 traces of the same case: σ is the *historical trace* and σ*′* is the *labeled trace*.

f: σ → σ^′^ with | {e′_i_|#_act_e′_i_ = a} |_∀ e′j ∈ σ′_ ≥ | {e_i_|#_act_e_i_ = a} |_∀ ei∈ σ_

The function *f* identifies the relevance levels of each stage in a trace. A stage can be divided into several phases with distinct levels of relevance. Therefore, the number of events that correspond to activity *a* in the trace σ is smaller or equal to the number of events that correspond to the activity *a* in trace σ^′^.

#### Example

**Figure 2:**
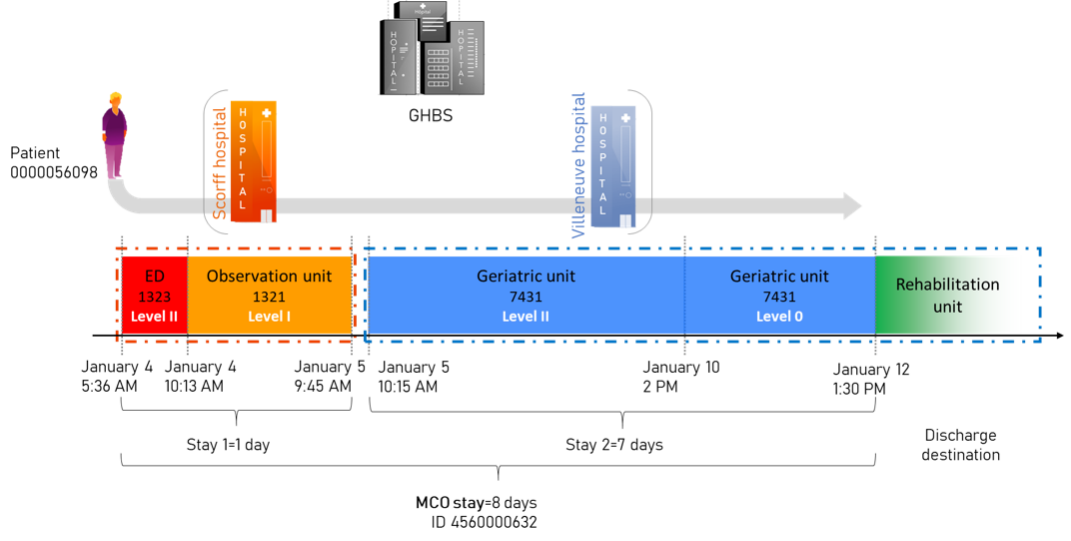
Illustration of the framework using a fictive pathway and patient. ED: emergency department; GHBS: Groupe Hospitalier Bretagne Sud; MCO: medicine, surgery, obstetrics, and odontology.

This paragraph illustrates the definitions and the transformation of a pathway using the function *f*. In the following fictive example, the hospital group is named Groupe Hospitalier Bretagne Sud (GHBS) and is composed of 2 sites, named Scorff and Villeneuve. One patient arrived on January 4 at 5:36 AM at the ED of the Scorff Hospital. At 10:13 AM, he was admitted to the observation unit (OU), but the patient was actually waiting for a bed in the geriatric unit. On January 5 at 9:45 AM, the patient was transferred to the geriatric unit of the Villeneuve Hospital, another site of the hospital group. He arrived at 10:15 AM. On January 10 at 2 PM, the patient was medically fit for discharge. On January 12 at 1:30 PM, the patient was discharged, and he returned home with additional community nursing services. Figure 2 illustrates the pathway of the patient according to the framework defined previously.

The pathway of patient *00000056098* can be formalized as follows:

σ =< e_11_, e_12_, e_21_, e_22_, e_31_, e_32_ > with

#*_activity_*(e_11_) = ED, #*_trans_*(e_11_) = start

#*_activity_*(e_12_) = ED, #*_trans_*(e_12_) = end

#*_activity_*(e_21_) = OU, #*_trans_*(e_21_) = start

#*_activity_*(e_22_) = OU, #*_trans_*(e_22_) = end

#*_activity_*(e_31_) = GERIATRICS, #*_trans_*(e_31_) = start

#*_activity_*(e_32_) = GERIATRICS, #*_trans_*(e_32_) = end

The function *f* takes the trace σ as input and returns the trace σ’ =< e’_11_, e’_12_, e’_21_, e’_22_, e’_31_, e’_32_ > with the following features:

#*_activity_*(e’_11_) = (ED, level2), #*_trans_*(e’_11_) = start

#*_activity_*(e’_12_) = (ED, level3), #*_trans_*(e’_12_) = end

#*_activity_*(e’_21_) = (OU, level1), #*_trans_*(e’_21_) = start

#*_activity_*(e’_22_) = (OU, level1), #*_trans_*(e’_22_) = end

#*_activity_*(e’_31_) = (GERIATRICS, level2), #*_trans_*(e’_31_) = start

#*_activity_*(e’_32_) = (GERIATRICS, level2), #*_trans_*(e’_32_) = end

#*_activity_*(e’_41_) = (GERIATRICS, level0), #*_trans_*(e’_41_) = start

#*_activity_*(e’_42_) = (GERIATRICS, level0), #*_trans_*(e’_42_) = end

The succession of stages is described by level-2 activity labels. The initial stage *Geriatrics* has been divided into a relevant phase (level 2) and an irrelevant phase (level 0).

### Automatic Pathway Labeling and Correction

#### Overview

**Figure 3:**
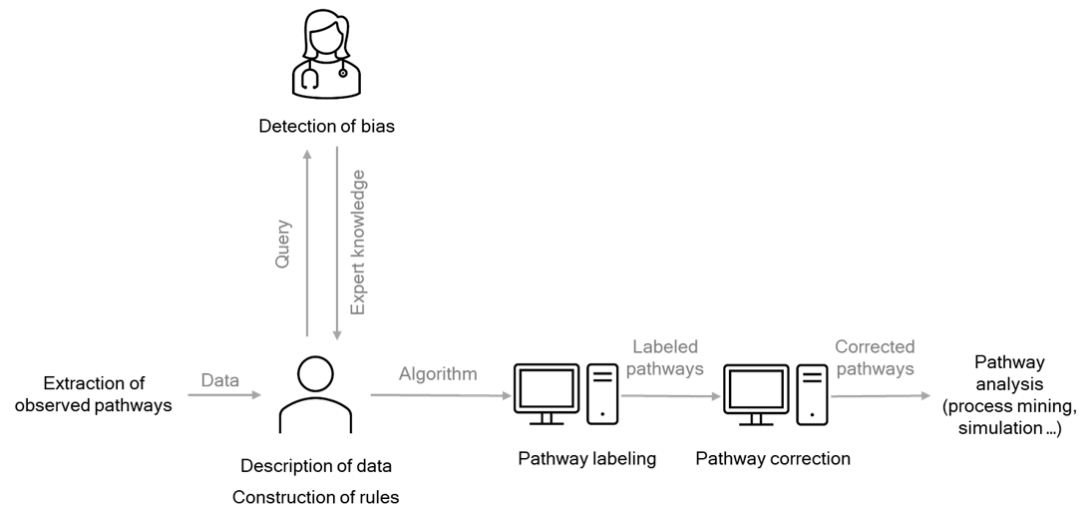
Pathway-labeling method.

In this section, the method for identifying bias in patient pathway data and the method for building an algorithm to label the stages of the pathways as relevant or irrelevant are described. We then present an algorithm for automatically transforming a historical trace into a labeled trace and an algorithm for automatically transforming a labeled trace into a corrected trace. These algorithms are based on a symbolic approach. In other words, the rules are *if, then* propositions. Our approach can be visualized in Figure 3.

#### Identification of Bias and Rule Definition

#### Overview

The methodology for identifying the bias and then defining the rules consists of three steps, which are described as follows:

1. Description of the pathway dataset: the objective was to distinguish the different pathways and identify frequent and rare patterns.
2. Identification of bias with expert interpretation: the objective was to analyze the patterns with experts to determine the bias.
3. Definition of rules: the rules are defined based on the experts’ analysis.

The first step can be achieved by computing the frequency and representativeness of each pathway variant and the number of events per variant, grouping some events, and computing the new variants to identify repetitive patterns. The second step enables us to identify bias through discussions with experts. Preferably, the discussions are conducted with several physicians to obtain different opinions. The third step requires the translation of the analyses of physicians into rules. A rule deduces whether an event is relevant from the EHR data. According to our definition of relevance (cf Definition 6), we consider three levels: (1) level 0, in which the stage is completely irrelevant (none of the conditions are met); (2) level 1, in which the stage is not totally relevant (only the first condition is met); and (3) level 2, in which the stage is relevant (both conditions are met). The aim of the rules is to identify in a pathway the phases with various levels of relevance. We remind the reader that only the hospitalization ward (patient location) is evaluated.

#### Example

A rule states that, if the first stage of the trace lasts <10 units, then the stage is completely irrelevant (level 0).

Let σ =< e_11_, e_12_, e_21_, e_22_ > be a trace with 2 activities a_1_ and a_2_ such that the following conditions are met: (1) #*activity*(e_11_) = #*activity*(e_12_)=a1; (2) #*activity*(e_21_) = #*activity*(e_22_)=a2; and (3) #*time*(e_11_)=0, (4) #*time*(e_12_)=7, and (5) #*time*(e_21_)=7*,* #*time*(e_22_)=20*.*

The first stage is a_1_ and lasts 7 units; therefore, e_11_ and e_12_ are labeled as level 0.

#### Algorithm 1: Pathway Labeling

Once the rules have been defined, algorithm 1 (Textbox 2) labels a pathway according to these rules (lines 6-9). A level of 1 or 0 is assigned if a stage is identified as irrelevant, and a level of 2 is assigned if the stage is relevant (lines 7-9). A stage can be labeled through several rules. In this case, the worst label is applied (ie, level 0 has a priority over level 1, and level 1 has a priority over level 2 [lines 10-12]).

We want to emphasize that this algorithm is purely based on logic and administrative rules. It does not include medical reasoning and is, therefore, inaccurate. However, the aim of the next section is to evaluate this inaccuracy (ie, the number of errors between an algorithm with simple rules and the complex reasoning of an expert [expert knowledge]).

**Algorithm 1—pathway labeling.**

1: Let *e_x_* be an event of the historical pathway

2: Let *e’_x_* be an event of the labeled pathway

3: Let *t*_1_ be the start date of the stay.

4: Let *t_n_* be the end date of the stay.

5: Let *σ =< e_11_, e_12_,..., e_n1_, e_n2_ >* be the trace representing the historical pathway.

6: Let L be a list that stores the result of each rule.

7: for each rule *r_k_* do

8: Add *r_k_*(σ) to L

9: end for

10: for each *e_x_* ∈ σ do

11: Apply the modification of each rule that has changed *e_x_*. The lowest relevance level has priority.

12: end for

13: Return *σ’ =< e’_11_, e’_12_,..., e’_m1_, e’_m2_ >*, the labeled trace.

#### Algorithm 2: Pathway Correction

We also implemented an algorithm to correct the pathways labeled using algorithm 1 (Textbox 3). The idea is to transform the observed pathway into a theoretical pathway by correcting irrelevant stages. The different corrections applied to a labeled pathway are deduced from the rules. The irrelevant activities are replaced with the relevant activities (lines 4-6). Only the label of an event is changed, and the time stamp remains the same. At the end of the correction, subsequent identical activities are merged (lines 7-13). For example, let us note a stage (activity name, relevance level, start, or end). The pathway *<(ED, level2, t1, t2)*, *(OU, level1, t2, t3)*, *(Geriatrics, level2, t3, t4)>* is corrected and becomes *<(ED, t1, t2)*, *(Geriatrics, t2, t3)*, *(Geriatrics, t3, t4)>.* In addition, the pathway can be merged to become *<(ED, t1, t2)*, *(Geriatrics, t2, t4)>*.

**Algorithm 2—pathway correction.**

1: Let *σ’ =< e’_11_, e’_12_,..., e’_m1_, e’_m2_ >* be the labeled trace.

2: Let *r* be the rule applied at *e’_x_*.

3: Let *σ’’ =< e’’_11_, e’’_12_,..., e’’_m1_, e’’_m2_ >* be a copy of σ*’*.

4: for each *e’’_x_* ∈ σ*’’* do

5: #*_activity_*(*e’’_x_*) = the corrected activity according to the rules of correction

6: end for

7: for each *e’’_x_* ∈ σ*’’* with t_x_ < t_m_ do

8: if #*_activity_*(*e’’_x1_*) = #*_activity_*(*e’’_x+1,1_*) then

9: #*_time_*(*e’’_x2_*) = #*_time_*(*e’’_x+1,2_*) with #*_trans_*(*e’’_x+1,2_*) = complete and all the events of stage *x+1* are deleted from σ*’’*

10: end if

11: end for

12: Return *σ’’ =< e’’_11_, e’’_12_,..., e’’_p1_, e’’_p2_ >*, the corrected trace.

#### Evaluation of the Performance of the Labeling Algorithm

This subsection describes the method used to assess the labeling algorithm. Because there is no reference to compare the results of the algorithm with a ground truth, the evaluation of the algorithm has to be made by comparing its results with the analyses of experts. The methodology used for this study is inspired by the framework developed by the French think tank Ethik IA for its humane oversight board (Ethik-IA, unpublished data, April 2021). A representative sample of patient pathways was analyzed by 2 experts. They had access to information from the electronic patient records. Each expert performed the analysis separately. The results of the first expert were compared with those of the second expert. When the results did not match, the medical experts discussed them to find a common answer. Their answers were then compared with the algorithms’ answers. For each difference, a discussion with the experts allowed us to determine whether the algorithm was wrong and, if so, qualify the errors.

The method presented in this paper is general and can be applied at any hospital. In the next section, we apply these methods to a real case study to create rules to label and correct a real dataset extracted from a hospital database, and we evaluate the accuracy of these rules.

### Ethical Considerations

This study was approved by the French Data Protection Authority (*Commission Nationale de l’Informatique et des Libertés*) under the number 922243. French and European rules about access to health care data for research were respected and ethical standards also.

## Results

### Data

This work was performed with the GHBS, a French hospital group located in the Lorient area. It has 2 general hospital sites with an ED and 6 other sites. In total, there were 89,791 ED visits and 108,875 hospitalizations and sessions (values for 2021). This study was based on data collected at the GHBS. Data were retrospectively collected for the period from July 2020 to July 2021. The data cover 12 months of activity and only adults, 54,850 different ED visits and 41,161 unique patients, including 19,905 MCO stays. Multiple MCO stays of the same patient were treated as separate instances. Three sources of data were used: (1) electronic patient records, (2) administrative health care databases, and (3) data from the software used for rehabilitation and home hospitalization. Only structured data were used to save time in the data analyses; no plug-and-play natural language processing tool was available for our data. The pediatric and obstetric pathways were excluded from the study dataset, as were the pathways with only a visit to the ED.

### Identification of Bias and Rule Definition

In this section, we detail the results obtained using our method to identify bias from our data.

#### Results of Step 1: Description of the Pathway Dataset

In our dataset, there were 19,905 pathways and 1013 trace variants. Some variants were very frequent, such as (ED, OU) representing 22.75% (4528/19,905) of the pathways, and others were very rare, such as (ED, neurology intensive care, cardiology) occurring just once.

We observed that most pathways had only 1 stage after the ED visit (60/1013, 5.92% of the variants and 15,699/19,905, 78.87% of the pathways). Pathways with >3 stages after the ED were rare. They appeared between 1 and 3 times in the dataset and represented 0.98% (195/19,905) of the pathways but 18.85% (191/1013) of the variants. Therefore, the diversity of pathways was mainly due to pathways with many activities. We identified five types of pathways at the GHBS: (1) mono-disease pathways, which include 1 necessary medical unit; (2) pathways for patients who were seriously ill, which include transfer to an intensive care unit (ICU); (3) older person pathways, which include geriatric units; (4) frequent and processed pathways, which include strokes; and (5) multi-disease and complex pathways, which include several medical units.

The most frequent medical units were OUs, polyvalent medicine units, geriatric medicine units, postemergency units, surgery units, and specialized medical units. By categorizing the medical units into 4 groups (ED, medicine, surgery, and ICU), we obtained 165 patterns, and the 10 most frequent structures of the pathways are listed in Table 1.

Within the pathways (MEDICINE, MEDICINE), several patterns were frequently observed. Pathways such as heart failure or stroke pathways were normally composed of 2 steps after the ED: admission to a cardiology (or neurology, respectively) ICU followed by cardiology (or neurology, respectively). The second type of pattern is the pathway with an admission to a polyvalent unit before an admission to a specialized unit or another polyvalent unit. In this case, a polyvalent unit is a medical unit, such as an OU, polyvalent medicine unit, or postemergency unit, where patients with multiple diseases or who do not require specialized treatment are treated. We also observed a few transfers between specialized units. Finally, patients could be transferred between the weekly hospitalization unit and the full hospitalization unit.

For most of the pathways that followed the pattern (SURGERY, MEDICINE), the activity of surgery was noted as an overflow bed. For the few others, a surgical act was performed before a transfer for medical reasons to a medicine unit.

The pattern (MEDICINE, SURGERY) mainly concerned an admission to an OU (while waiting for surgery) followed by a surgery unit. In the other pathways, patients were first admitted to a specialized unit (eg, hepatogastroenterology) before surgery.

Hence, we obtained four patterns for the pathways in 2 steps: (1) a polyvalent unit followed by a specialized unit (pattern 1), (2) a surgery unit followed by a medical unit (pattern 2), (3) a daily or weekly hospitalization unit followed by a full hospitalization unit of the same specialty (pattern 3), and (4) a specialized unit followed by another specialized unit (pattern 4).

**Table 1:** Main structures of historical pathways and occurrence differences for corrected pathways.

Variant	Occurrence (%)	
	Historical	Corrected
ED^a^, MEDICINE	68.68	68.57
ED, MEDICINE, MEDICINE	10.67	7.24
ED, SURGERY	9.66	10.90
ED, SURGERY, MEDICINE	2.88	0.33
ED, MEDICINE, SURGERY	1.7	0.63
ED, MEDICINE, MEDICINE, MEDICINE	1.2	0.80
ED, ICU^b^, MEDICINE	0.88	1.05
ED, ICU	0.53	0.56
ED, SURGERY, SURGERY	0.47	0.16
ED, ED, MED	0.39	0.22
ED	—^c^	7.18

^a^ED: emergency department.

^b^ICU: intensive care unit.

^c^Not present in historical pathways.

#### Results of Step 2: Interpretation of Patterns by Experts

We discussed the patterns identified in the first step with the experts. According to the experts, pattern 1 (a polyvalent unit followed by another unit) usually indicates that the polyvalent unit is used as a buffer. In fact, when beds are lacking, patients can be admitted to a polyvalent unit to begin their treatment while waiting for a bed in the ideal unit. The second pattern has the same explanation—a patient requiring medical treatment is placed in a surgery unit while waiting for a bed in the ideal unit. Pattern 3 (transfers between daily or weekly hospitalization units and full hospitalization units) is explained by a lack of beds in the full hospitalization unit. The last pattern (transfers between specialized units) has no general explanation. Occasionally, a specialized unit can also be used as a buffer, but the transfer can also be medically explained.

The length of stay (LoS) was also an aspect of the pathway discussed with the experts. Some pathways are too long because patients cannot be discharged as soon as they are medically fit for MCO discharge. The delay is mainly due to a back home impossible without community care or a lack of beds in rehabilitation centers. LoS can be compared with a national reference. In France, the national reference is the average LoS used for hospital stay invoicing (*durée moyenne de séjour* in French). It is defined for each diagnosis-related group. However, each pathway is unique, and an LoS above the national reference does not guarantee that the stay was too long because of discharge difficulties. For example, longer stays for patients receiving palliative care are frequent and normal. Occasionally, the diagnosis-related group does not correctly report the seriousness of the patient because the patient was transferred to another hospital.

The LoS in the ED was also discussed. According to the experts, the LoS in the ED is occasionally too long because patients are waiting to be hospitalized. The ED LoS should not exceed 5 to 10 hours.

#### Results of Step 3: Rule Construction

From these observations and discussions, we deduced several dimensions to investigate in a pathway:

1. Time spent in the ED: ED LoS can be prolonged because of a lack of inpatient beds in acute care units. Rule 1 evaluates whether the time spent in the ED is too long.
2. LoS: the LoS can be prolonged because of a delayed discharge. Rules 2 and 3 evaluate this condition.
3. Overflow bed: occasionally, patients are admitted to a medical unit but are treated by the physicians of another unit. The location of the patient is entered (ie, the activity name) in the hospital data, and the unit medically responsible for the patient is also indicated. For example, when a patient is in surgery and awaits a bed in cardiology, the activity is surgery and the medically responsible unit is cardiology.
4. Sequence of activities: a typical pattern is the transfer between a polyvalent unit and a specialized unit. According to physician experience, when the transfer occurs within 1 week, in general, the polyvalent unit stage is irrelevant. In rule 5, the threshold is 7 and a half days to consider the transfer time. Rule 6 is dedicated to the OU because, in our dataset, a transfer in the OU is labeled as “back home within 24 h,” “observation,” or “awaiting bed.” The label “awaiting bed” indicates an irrelevant stage because the patient should have been transferred immediately to the appropriate unit.

The challenge was to identify which structured data could be used to investigate these different dimensions and, therefore, to create the rules. We detail the rules obtained from our dataset in Multimedia Appendix 2, as well as the algorithms of the 7 rules.

From these rules, we deduced how to correct the labeled pathways. The different corrections are listed in Table S1 in Multimedia Appendix 2.

#### Example

**Figure 4:**
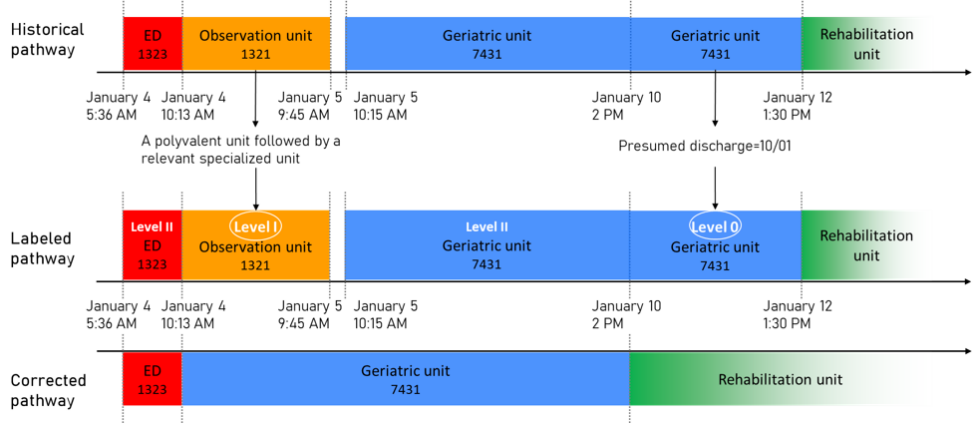
Example of the correction of a pathway. ED: emergency department.

Figure 4 illustrates the labeling and correction of a pathway. In total, 2 phases are considered irrelevant by the algorithm: the OU (rule 6) and the end of the stay in the geriatric unit (rule 3). To correct the pathway, the time spent in the OU is replaced by the time spent in the geriatric unit, which is the relevant stage following the OU, and the patient is discharged earlier at the presumed discharge date.

### Accuracy of the Labeling Algorithm

A total of 118 different pathways were analyzed by 6 different duos of physicians from the GHBS (only 1/12, 8% had previously participated in the discussion to define the rules) and compared with the algorithm output. Only rules 3, 4, 5, 6, and 7 could be evaluated because the physicians could not reach a consensus on either the ideal LoS or the ideal length of ED visit within the allotted time. The physicians had access to the patient’s pathway, their age, the chief complaint, the diagnoses, the national LoS reference for the diagnosis-related groups, the discharge destination, and requests for rehabilitation and home hospitalization. They also had access to patient records if more information was needed.

We counted the number of errors per stage (except for the first ED visit) and per pathway. For example, the pathway (ED, OU, CARDIOLOGY) has 2 stages (we do not count the ED), and the experts assessed that OU was not relevant and that CARDIOLOGY was relevant, but the algorithm assessed that the 2 stages were relevant. Therefore, we identified 1 pathway error per 1 pathway and 1 stage error per 2 stages. Table 2 presents the results of the evaluation of the 118 pathways. The algorithm failed to evaluate 19.5% (23/118) of the pathways and 12.9% (30/232) of the stages. Among these errors, false-positive errors were 5 and 3 times more important, respectively, than false-negative errors. This means that the main error of the algorithm was considering a pathway or a stage as relevant when it was actually irrelevant.

The main errors were related to medical knowledge. When patients were hospitalized in a specialized medical unit (eg, oncology) but pertained to another specialized medical unit (eg, cardiology), the algorithm did not detect this irrelevance. Similarly, hospitalization in the general ICU before transfer to the cardiology ICU was occasionally irrelevant but not detected by the algorithm. In addition, the algorithm considered that a polyvalent unit following a specialized unit was irrelevant; however, in some cases, it was wrong.

**Table 2:** Performance of the algorithm based on rules 3, 4, 5, 6, and 7.

				Actual values					Precision				Recall	
				Positive ^a^		Negative ^b^								
**Pathways**										0.75				0.93
	**Predicted values**													
		Positive	56		19									
		Negative	4		39									
**Stages**										0.88				0.95
	**Predicted values**													
		Positive	162		22									
		Negative	8		40									

^a^Relevant pathway or stage (level 2).

^b^Irrelevant pathway or stage (level 0 and level 1).

### Preprocessing of Pathway Data

In this section, we use only rules 3 to 7 to preprocess our data given that rules 1 and 2 could not be assessed. We evaluated 19,832 pathways, including 24,989 stages (excluding the first ED stage). Of these 19,832 pathways, 2669 (13.46%) were evaluated as irrelevant, and 11.21% (2802/24,989) of stages were also evaluated as irrelevant. Considering the error margin, between 2162 and 3176 pathways were irrelevant, and between 2438 and 3166 stages were irrelevant. The main irrelevant movements detected by the algorithm were overflow bed in surgery, overflow bed in polyvalent units, and waiting time in the OU. The 19,832 historical pathways included 986 (4.97%) variants. Once corrected, 792 variants were noted. Table 1 details the distribution of the structures of the variants. We observed that the *ED, MEDICINE, MEDICINE* and *ED, SURGERY, MEDICINE* traces were less frequent than before correction, which is due to the correction of the overflow beds. The trace ED appeared because the OU stage was assessed as irrelevant for several *ED, OU* (included in *ED, MEDICINE*) traces.

### Statistical Analysis of Relevant and Irrelevant Pathways

Once the pathways were labeled, we compared the relevant pathways with the irrelevant pathways to understand the causes of irrelevance in the pathways. Several causes were already known among medical and administrative staff: bed occupation rates, ED crowding, age, and discharge destination. We tested these hypotheses using 4 bivariate analyses. The 4 variables to explain were an ED LoS of >5 hours, an ED LoS of >10 hours, the presence of overflow beds, and delayed discharge. For categorical variables, the proportions were compared using a chi-square test. For quantitative variables, the distributions were compared using a 2-tailed Student *t* test. We studied different explanatory variables: weekday corresponds to the start of the ED visit or admission to the inpatient unit; the arrival period is divided into 4 periods (morning from 7 AM to noon, afternoon from noon to 5 PM, night from 5 PM to 11 PM, and deep night from 11 PM to 7 AM); the next historical stage is the inpatient unit where the patient was admitted, and the next corrected stage is where the patient should have been admitted (based on the evaluation of the pathways); the last stage is the medical unit from which the patient was discharged; and the ED crowds are the number of patients present in the ED when the patient arrives.

Table 3 reports the bivariate analyses. Tables S2 and S3 in Multimedia Appendix 3 provide the detailed results. Several observations can be made from the statistical analysis. First, the seasons and the irrelevance of the next stage are not significant for a delay in the ED of >5 hours, but they are significant for a delay of >10 hours. Second, counterintuitively, fewer irrelevant post-ED admissions occur for long ED delays. This finding is probably because patients who stay in the ED for a long time are ultimately admitted to a relevant unit. Third, the weekday of patient arrival influences the ED delay. On Mondays, more patients wait >5 hours, and the proportion decreases throughout the week and increases again on Sundays. This phenomenon is caused not only by the greater number of patients admitted on Monday but also by the difficulty in hospitalizing patients during the weekend. Therefore, on Sunday, many patients are waiting for hospitalization. Fourth, this is the same observation and explanation for the overflow beds; more patients are admitted to an irrelevant unit on Sunday. Fifth, crowding in the ED was less important for the longest ED delays. Indeed, patients arriving at night or late at night are less likely to be transferred to an inpatient unit, and this is also the period during which fewer patients arrive at the ED. Sixth, a greater number of patients are admitted to irrelevant units when the ED is more crowded. Seventh, increased age is a factor of long delays in accessing the ED. Eighth, similar features are noted for the occupation rate of the next stage. Ninth, the next corrected stages had an occupation rate (95%) higher than that of the next historical stages (92%). Tenth, age does not impact the risk of being in an overflow bed. Eleventh, the season has an impact on discharge delays. Specifically, in summer, more discharges are delayed, perhaps because the health care supply is lower during the summer holidays. Twelfth, the proportion of delayed discharges varies according to the destination of the discharge. Discharges at a psychiatric center have the highest rate of delay (97/203, 47.8% of delayed stays), followed by discharges at rehabilitation centers (1285/3294, 39.01% of delayed stays). Delayed discharges for death correspond to requests for palliative care at home or at another center that were not accepted in time. Thirteenth, age does not affect the risk of delayed discharge.

The period of study was impacted by the COVID-19 pandemic. These results could be more robust with access to a longer period of study (3 years instead of 1), and the seasons variably could be assessed several times during a longer time frame. Furthermore, the quality of the data was imperfect, especially for the computation of the occupation rate. Therefore, the results should not be extrapolated to other periods or hospitals. However, the analysis allowed us to compare the relevant and irrelevant pathways because they were derived from the same dataset. Hence, we can conclude that significant differences (*P*<.001 for most of the features) were observed between relevant and irrelevant pathways. Logistic factors such as the day of the week, the hour of arrival, medical unit occupation, and the discharge destination influence the risk of overflow.

**Table 3:** Bivariate analysis.

Variable to explain and features			*P* value
**ED^a,b^ visit of >5 h**			
	Age	<.001	
	ED crowds	<.001	
	Occupation rate historical next stage^c^	<.001	
	Occupation rate corrected next stage^c^	<.001	
	Weekday	<.001	
	Season	.51	
	Arrival period	<.001	
	Next stage irrelevant	.05	
	Next historical stage	<.001	
	Next corrected stage	<.001	
**ED^a^ visit of >10 h**			
	Age	<.001	
	ED crowds	<.001	
	Occupation rate historical next stage^c^	<.001	
	Occupation rate corrected next stage^c^	<.001	
	Weekday	<.001	
	Season	<.001	
	Arrival period	<.001	
	Next stage irrelevant	<.001	
	Next historical stage	<.001	
	Next corrected stage	<.001	
**Overflow beds**			
	Age^d^	.03	
	ED crowds^e^	<.001	
	Occupation rate corrected unit^f^	<.001	
	Arrival hour^f^	.37	
	Weekday	<.001	
	Season	.78	
	Arrival period	<.001	
**Delayed discharge**			
	Age	.72	
	Discharge destination	<.001	
	Last stage	<.001	
	Season	<.001	

^a^The analysis was performed exclusively using the data from the principal site (Scorff) because emergency department crowds and age differ between the principal site and the smaller site (Villeneuve).

^b^ED: emergency department.

^c^The next historical stage is the inpatient unit where the patient was admitted, and the next corrected stage is where the patient should have been admitted (based on the evaluation of the pathways). Occupation rate is the number of patients already present in the unit over its capacity.

^d^We compared the set of pathways without an overflow stage and the set of pathways with at least one overflow stage.

^e^In each pathway, the ED crowds were only computed for the first medical unit subsequent to the ED stage.

^f^We compared the sets of relevant and irrelevant stages.

### Synthesis of Patient Pathway Labels

To summarize this section, from the analysis of the structure of the patient pathways and expert knowledge, we built 7 rules that detect irrelevant stages in a patient pathway (description of the dataset and construction of rules). On the basis of these rules, we used a pathway-labeling algorithm that labels the stages of a pathway according to 3 levels of relevance (pathway labeling). We then used a pathway correction algorithm that transforms a labeled pathway into an ideal pathway (pathway correction). The evaluation of our algorithm showed that it exhibits 87% accuracy. In our dataset, 13.46% (2669/19,832) of the pathways were labeled as irrelevant. Finally, a statistical comparison between relevant and irrelevant pathways demonstrated that logistic constraints influence the quality of patient pathways.

The next 2 sections show the importance of this preprocessing step before analyzing patient pathways using process mining and of using these data for hospital management.

### Case Study 1: Analysis of Patient Pathways Using Process Mining

#### Motivation

The first case study investigates the impact of our preprocessing technique on process discovery. We evaluated the ability of our preprocessing method to simplify process graphs. We compared the process graph of an event log comprising historical traces with the process graph of an event log comprising corrected traces. We used the ProM framework (version 6.12; ProM Tools) [38] to discover the graphs and estimate different metrics. The process graphs were computed using the Fodina algorithm [39], which outputs a causal graph that was transformed into a Petri net with the plug-in “Convert Causal net (C-Net) to Petri net” (F. Mannhardt).

#### Metrics

The metrics were computed using the plug-in “Show Petri-net Metrics” (HMW Verbek). This plug-in computes (1) the extended Cardoso metric (ECaM), (2) the extended Cyclomatic metric (ECyM), (3) the structuredness [40], and (4) the density [41].

The ECaM counts the splits (XOR, OR, or AND) in the net and penalizes each of them. The ECyM is the difference between the number of edges and vertices plus the number of strongly connected components. According to Lassen and Van der Aalst [40], a high ECaM score can be caused by a “high degree of fan-out from places,” and numerous parallelisms can increase the ECyM. Structuredness recognizes different types of structures and scores each structure by giving it a penalty value. Finally, the density relates the number of arcs to the number of all possible arcs for a given number of nodes. Therefore, these 4 metrics quantify the different structural characteristics of a graph.

#### Quantitative Analysis

The process graph discovered from the whole dataset of pathways is spaghettilike because the number of variants is large with or without correction (986 vs 792). To avoid spaghettilike effects, we reduced the analysis to 1 medical unit (ie, the event log included only the traces that contained this activity). Table 4 shows the metrics calculated for 5 medical units. As expected, the corrected event log contains fewer variants and activities than the historical event log. Consequently, the number of arcs, places, and transitions on the graph also decreases. The historical graph density is greater than the corrected graph density. This finding indicates that the corrected graph is more compact than the historical graph. The ECaM and ECyM of the corrected graph are lower than those of the historical graph. Indeed, we can observe more places with many output transitions and more parallelisms in the historical Petri net. Finally, the structuredness of the historical graph is also greater than the structuredness of the corrected graph except for the neurology unit. This means that more complex structures or more unstructured components are observed in the historical graphs than in the corrected graph. The neurology exception can be explained by the fact that a state machine is identified in the historical graph but only an unstructured component is identified in the corrected graph. In conclusion, the corrected Petri nets can be considered simpler than the historical Petri nets.

**Table 4:** Comparison of the historical and corrected process graph.

Process graph		Variants, N	Activities, N	Arcs, N	Places, N	Transitions, N	Density	ECaM^a^	ECyM^b^	Structuredness
**Cardiology**										
	Historical	13	10	32	7	16	0.14	11	16	64
	Corrected	9	7	22	6	11	0.17	8	11	22
**Visceral surgery**										
	Historical	19	15	42	8	21	0.13	12	20	42
	Corrected	8	6	20	6	10	0.17	8	10	20
**Polyvalent medicine**										
	Historical	12	13	76	16	38	0.06	33	27	76
	Corrected	7	6	28	9	14	0.11	13	11	56
**Geriatric medicine**										
	Historical	5	6	14	4	7	0.25	3	7	9.5
	Corrected	3	4	14	4	5	0.25	3	5	6.5
**Neurology**										
	Historical	24	13	52	11	26	0.09	21	26	208
	Corrected	16	8	37	10	18	0.10	17	24	1602

^a^ECaM: extended Cardoso metric.

^b^ECyM: extended Cyclomatic metric.

#### Qualitative Analysis

A qualitative analysis can also be performed (see Multimedia Appendix 4 for the pictures of the graphs). The cardiology historical graph (Figure S1 in Multimedia Appendix 4) shows that several activities can occur before admission to cardiology, but it is not easy to distinguish between groups of patients. The corrected graph is easier to read, and four groups of patients can be identified: (1) serious patients who need intensive or continuous care before being admitted to cardiology, (2) patients who need to be permanently monitored for cardiac examination, (3) patients who need pulmonology care before cardiology care, and (4) patients who do not need other care before transfer and are directly admitted to cardiology (with eventually a step in the OU before).

The historical graph of the polyvalent medicine unit (Figure S2 in Multimedia Appendix 4) is complex to read, and several specialized units are present but not related to the polyvalent unit in the graph. The corrected graph is much simpler to read. Three groups of stays are identified: (1) stays with intensive or continuous care before admission to polyvalent medicine, (2) stays with direct admission, and (3) stays with a step in the OU or seasonal unit before admission to polyvalent medicine.

Equivalent analyses can be performed on other medical units (Figures S3, S4 and S5 in Multimedia Appendix 4). The neurology graph (Figure S3 in Multimedia Appendix 4) is less simple than the other graphs, possibly because patients going to neurology have complex pathways or because the correction of neurology pathways requires particular rules. However, the corrected graph is again more interpretable than the historical one.

### Case Study 2: Estimation of Ward Capacity Through Simulation

#### Motivation

Computer simulations can be used to estimate the number of beds necessary in each medical unit to admit unscheduled patients and help solve capacity planning problems. Indeed, the actual number of patients admitted to each medical unit does not include all the patients not admitted because of a lack of beds, and the number of the patients who should not be admitted to each unit is considered. Hence, it does not reflect the real need for beds. To solve this problem, pathway correction can be used. This method can be useful for estimating capacities when building or renovating a hospital or for organizing medical teams.

#### DES Model

**Figure 5:**
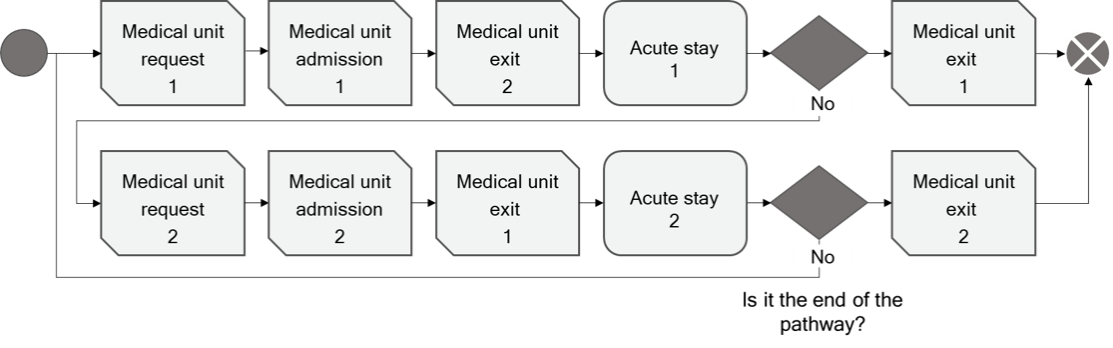
Modeling of patient flows through medical units.

In this case study, we simulate patient flow through the medical units of one hospital to compute the level of occupation of the medical units. We compared a simulation with the historical pathways (scenario 1) and a simulation with the corrected pathways (scenario 2). To do so, we modeled the medical units and patient flow using the AnyLogic software (The AnyLogic Company). Figure 5 shows the DES model.

The model represents 1 general hospital and 20 full hospitalization units. Each patient has a succession of units to follow. The model simulates the admissions of patients to medical units, their stays, and their discharges. A patient exits the simulation when they have completed their pathway. To simulate the source of patients, we used the dataset described in section Data (only patients from the main general hospital site [named Scorff] were included). We filtered the dataset based on the following criterion: only the pathways with at most 3 activities (ED visits plus 1 or 2 units) were included. We excluded pathways with weekly or daily units, the rare variants (coverage percentage of <0.001), and patients from sites other than Scorff. The corrected pathway dataset was filtered to keep only the stays included.

#### Experimental Settings

The capacities of the medical units were set to infinity to calculate how many unscheduled patients needed to be admitted to each unit each day. The LoS in each unit was randomly generated according to a probability law. To choose this probability law, we compared several distributions (normal, β, γ, log-normal, Weibull, and exponentiated Weibull) and fitted them on the stays of our dataset (between 150 and 5000 stays per medical unit). The log-normal distribution best fit the LoSs in each unit. The parameters of the log-normal distributions were adjusted for each unit by fitting the distribution to the real values. The log-normal distribution tends to generate more extreme values than those observed in reality; thus, the LoS was limited to 28 days in a unit and 24 hours in the ED. The simulated patients’ arrival dates were fixed and equal to the real patients’ arrival dates. The simulated patients either followed the historical pathways (scenario 1) or the corrected pathways (scenario 2). The simulation duration was 1 year, and the simulation run included 13,366 pathways.

To choose the warm-up time, we monitored the mean number of present patients in each medical unit over the simulation time. After 50 days, a stable situation was reached (Figure S6 in Multimedia Appendix 5). The warm-up time was set to 2 months. The number of replications was chosen to have an error of <10%. In total, 15 replications allowed this target to be reached and a reasonable simulation time to be reached—approximately 1 minute is required for 1 run, and the 15 replications take 10 minutes. The results are the mean values of 15 replications.

The simulation model was validated by comparing the mean LoS of each medical unit from the simulation results to those from the real dataset. This was the only source of randomness in the model as the simulated patients arrive according to the real dataset and follow a deterministic pathway. The mean absolute error was 0.6 days, which is <10% of the mean length of a hospital stay (Table S4 in Multimedia Appendix 5).

#### Results of DES

Table 5 shows the mean number of acute patients present in each unit in both scenarios. For several units, the number of patients differed between scenario 1 and scenario 2. For example, the polyvalent medicine unit had, on average, 2 patients less with corrected pathways, and the neurovascular unit had 1 more patient. We also observed that, globally, there were fewer patients present at the same time with the corrected pathways compared with the historic pathways. Indeed, there were fewer stages in the corrected pathways because some were judged as irrelevant; therefore, in the simulation, the patients stayed less time in the hospital.

In conclusion, preprocessing pathway data is important for addressing capacity planning problems in hospitals. In this example, we observed that using historic pathways can lead to biased numeric interpretations for the capacity planning of medical units.

**Table 5:** Mean number of acute patients in medical units over 1 year.

Medical unit	Scenario 1	Scenario 2	Difference
3O surgery^a^, mean (95% CI)	7.6 (7.3-7.9)	1.1 (0.9-1.2)	–6.5
Orthopedic surgery, mean (95% CI)	10.1 (9.9-10.3)	13.3 (13.0-13.5)	3.1
Visceral surgery, mean (95% CI)	6.5 (6.0-7.1)	5.9 (5.4-6.3)	–0.7
Pulmonology, mean (95% CI)	14.2 (14.0-14.4)	14.3 (14.1-14.6)	0.1
Cardiology ICU^b^, mean (95% CI)	2.0 (1.8-2.2)	2.0 (1.8-2.2)	0.0
Cardiology, mean (95% CI)	10.1 (9.8-10.3)	10.1 (9.9-10.3)	0.1
Postemergency unit, mean (95% CI)	17.0 (16.7-17.3)	14.5 (14.3-14.7)	–2.5
Polyvalent medicine, mean (95% CI)	35.5 (35.5-35.5)	33.9 (33.9-33.9)	–1.7
Neurology ICU, mean (95% CI)	3.0 (2.7-3.3)	3.0 (2.7-3.3)	0.0
Neurovascular, mean (95% CI)	3.9 (3.8-4.1)	5.1 (4.8-5.5)	1.2
Neurology, mean (95% CI)	3.7 (3.7-3.7)	3.1 (3.1-3.1)	–0.7
Hepatogastroenterology, mean (95% CI)	13.3 (12.9-13.6)	13.7 (13.5-13.8)	0.4
Rheumatology, mean (95% CI)	11.4 (11.2-11.6)	11.3 (11.3-11.3)	–0.1
Observation unit, mean (95% CI)	8.7 (8.4-9.0)	6.5 (6.1-6.9)	–2.2
Geriatric medicine, mean (95% CI)	39.3 (39.0-39.5)	38.7 (38.5-38.9)	–0.5
ICU, mean (95% CI)	1.8 (1.5-2.1)	2.0 (1.7-2.3)	0.2
Seasonal unit^c^, mean (95% CI)	2.9 (2.5-3.2)	2.0 (1.7-2.3)	–0.9
Oncology hematology, mean (95% CI)	5.0 (4.7-5.3)	5.2 (4.8-5.6)	0.2
Nephrology endocrinology, mean (95% CI)	3.5 (2.9-4.0)	3.4 (3.0-3.8)	–0.1
CCU^d^, mean (95% CI)	0.6 (0.3-0.9)	0.6 (0.3-0.9)	0.0
Total patients, N	200	190	–10

^a^Ear, nose, and throat; ophthalmologic; and orthopedic surgery.

^b^ICU: intensive care unit.

^c^The seasonal unit is only open during the winter months. Therefore, the occupation figures computed over a year do not reflect reality.

^d^CCU: continuing care unit.

## Discussion

### Principal Findings

A framework and a methodology to study patient pathways were presented in this paper. They were used to develop a pathway-labeling algorithm that automatically detects whether a patient pathway is irrelevant (ie, contains stages due to resource limitations [as defined in the *Definition 6: Relevance of Stage* section]). Two main methods are available to achieve such a task: (1) building a thesaurus with medical experts (or using supervised learning) that links the main diagnosis (or the chief complaint) with an ideal pathway and (2) building a symbolic algorithm. The first method is the most accurate but is very time-consuming. This method would require hours of work with experts to build a thesaurus or annotate data for training a machine. None of these methods provide general results because the thesaurus and rules need to be adapted to each hospital. We chose the second option, an algorithm based on logic and administrative data, because it can be built quickly and is easily adaptable to organizational changes. We provided a general method to build this algorithm. We applied our algorithm to our dataset, and we were able to estimate the gap between our algorithm and an expert assessment. Our results demonstrate that a nonnegligible gap exists (13% to 19% of errors); however, we believe that the error rate was small enough for globally evaluated pathways. The estimation of this error also enabled us to identify the source of errors of the algorithm. On the basis of this labeling, a correction of the pathways was then performed to represent pathways that would be considered ideal.

We also demonstrated that resource limitations impact the choice of pathway using a statistical analysis that compared relevant and irrelevant pathways. The factors identified as increasing difficulties in managing patient flows could be included in hospitals’ strategies to improve patient pathways.

The 2 case studies illustrate the importance of preprocessing patient pathway data before any analysis. Studying and representing patient pathways using process mining is complicated (*Related Work* section). By focusing on pathways with a common medical unit, we demonstrated that a corrected graph is more interpretable than a historical graph. Hence, our algorithm is an efficient preprocessing tool for the analysis of patient pathways using process mining. The simulation of patient pathways is useful for testing bed management changes, but numeric results can be false if the input data include bias. In our example, the determination of the mean number of beds required for acute patients differed for the historic and corrected pathways. Some medical units need fewer beds, and others need more beds.

Pathway labeling should be applied before any analysis, such as process mining (case study 1), simulation (case study 2), or training of machine learning models to predict hospital pathways. In another work, we studied the prediction of the medical unit where a patient will be admitted after an ED visit. If raw data are used to train a machine learning model, the training will be biased. Indeed, the model will learn, for example, that some patients who do not need surgical treatment should be transferred to surgery. In contrast, if the model is trained using relevant pathways, it will learn the ideal medical unit for the patients [42].

### Limitations

We proposed a general method to study patient pathways and identify bias in the data. However, our approach could only be tested in 1 dataset because of legal constraints. Therefore, additional studies with other hospitals should be performed to validate the generalizability of our approach. Our labeling algorithm is not 100% accurate. To avoid errors, more rules could be created by exploiting textual data using natural language processing. Indeed, to build our algorithm, we only used structured data because of the unavailability of an adequate tool to treat textual data in our hospital.

### Conclusions

This work suggests a new approach to preprocess data on pathways of unscheduled patients. To our knowledge, there are no other studies that have evaluated nonspecific disease pathways. Our approach has the advantages of being explicable, simple to implement, and adaptable to each hospital.

Future research could develop process discovery techniques that consider the relevance labels of the activities.

## Appendix

Multimedia Appendix 1 Additional definitions.

Multimedia Appendix 2 Algorithms of the rules.

Multimedia Appendix 3 Satistical analysis results.

Multimedia Appendix 4 Process models.

Multimedia Appendix 5 Simulation parameterization.

## Abbreviations

DES: discrete event simulation
ECaM: extended Cardoso metric
ECyM: extended Cyclomatic metric
ED: emergency department
EHR: electronic health record
GHBS: Groupe Hospitalier Bretagne Sud
ICU: intensive care unit
LoS: length of stay
MCO: medicine, surgery, obstetrics, and odontology
OU: observation unit
