# Supplementary material for: Evaluating the Bias in Hospital Data: Automatic Preprocessing of Patient Pathways Algorithm Development and Validation Study
Source: JMIR Med Inform. 2024 Sep 23;12:e58978. doi: 10.2196/58978 (PMC11459108; doi:10.2196/58978)
Supplement: Multimedia Appendix 1 [file medinform_v12i1e58978_app1.pdf]

# Appendix 1: Additional Definitions

**Definition** (*Medical Unit*)**.** A medical unit provides treatment and care to inpatients and outpatients. We distinguish three main disciplines:

- Medicine, Surgery and Obstetrics units (MCO): These units provide acute treatments and care in medicine, surgery, obstetrics, odontology, and oncology.
- Rehabilitation units (Rehab): These units provide care for patients who need rehabilitation of a wound organ or who need to be readjusted to limited capacity.
- Psychiatry Units (PSY): These units provide care and treatment for patients with mental health disorders.

A medical unit is defined by a name, an id, a location (the hospital site where the medical unit is) and a discipline as follows:

mu = (name, id, site, discipline)

**Definition** (*Hospital*)**.** A hospital ensures the diagnosis, monitoring and care of sick people, injured people, and pregnant women.

**Definition** (*Hospital Group*)**.** A hospital group is a legal institution that includes different hospitals and residential care facilities. Therefore, a hospital group has multiple sites, and a site corresponds to a geographical location.

**Definition** (*Hospital Stay*)**.** A hospital stay is the period of time an inpatient has spent in the same hospital (site) and in medical units of the same discipline. In the healthcare records each stay is identified by an id. Hence, each time a patient is transferred from one site to another site or hospital, the stay id changes. Similarly, if a patient is transferred from one discipline (e.g. MCO) to another discipline (e.g. PSY), the stay id changes.

**Definition** (*Length of Stay (LoS)*)**.** The length of stay is the duration of a hospital stay. The LoS is generally expressed in days.

**Definition** (*Discharge from Hospital*)**.** The discharge is the official release from hospital care or from a medical care facility. The discharge disposition is the place where patients go after discharge.

The following definitions are specific to our framework.

**Definition** (*MCO-stay*). In this work, we need to enlarge the notion of a hospital stay to the hospital group. We considered the stays of patients through all the MCO units of the entire hospital group. Therefore, a stay begins when a patient is admitted to one of the MCO units of the hospital group and ends when the patient is discharged from the last MCO unit or transferred outside the hospital group.

**Definition** (Discharge from MCO dispositions)**.** We consider seven main types of discharge disposition after an MCO stay:

- Return home
- Return home with the help of community nursing
- Home hospitalisation: this service of home health care service provides complex and medical care at home by preventing patient from being at the hospital
- Transfer to a rehabilitation facility (rehab)
- Transfer to a psychiatric unit or a psychiatric hospital (PSY)
- Admission to a long-term care facility such as a nursing home
- External transfer: transfer to an MCO unit of another hospital group

Some dispositions require the development of an individual health care plan; and therefore discharge needs to be planned by medical and social staff.

**Example.** One hospital group had two general hospitals (sites), hospital A and hospital B. Patient Y visited the ED of hospital A. Then patient Y was admitted to the cardiology unit of the same hospital. After he or she was admitted to the geriatric unit of hospital B. Patient Y went to the

rehabilitation unit of hospital B. The patient visited 3 MCO units, 1 Rehab unit, and two hospitals of the same hospital group. The MCO-stay is the period of time from admission to the ED to discharge from the geriatric unit: ED → Cardiology → Geriatrics.
