# Supplementary material for: Evaluating the Bias in Hospital Data: Automatic Preprocessing of Patient Pathways Algorithm Development and Validation Study
Source: JMIR Med Inform. 2024 Sep 23;12:e58978. doi: 10.2196/58978 (PMC11459108; doi:10.2196/58978)
Supplement: Multimedia Appendix 2 [file medinform_v12i1e58978_app2.pdf]

Appendix 2: Algorithms of the Rules

Rule 1: If the ED stage is longer than the reference duration (e.g., 5 hours), then the stage is split into two stages: a stage (ED, level 2) from admission to + 5 h and a stage (ED, level 1) from + 5 h to discharge from the ED.

The only necessary data were the date of ED admission and the date of ED discharge.

Algorithm 3 Rule 1: Evaluation of the ED length of stay.

Let $e_{11} = (ED, start, t_1)$ and $e_{12} = (ED, end, t_2)$ be the two events of stage s_1.
if $\#_{duration}(s1)$ > 5h40 **then**
 $e'_{11} = ((ED, level2), start, t_1)$
 $e'_{12} = ((ED, level2), end, t_1 + 5h)$
 $e'_{21} = ((ED, level1), start, t_1 + 5\text{h})$
 $e'_{22} = ((ED, level2), end, t_2)$
end if

Rule 2: If the stay is longer than the time reference, then the stage that begins before the time reference and ends after the time reference is split into two stages: one stage (activity, level 2) from the start of the initial stage to the time reference and one stage (activity, level 0) from the time reference to the end of the initial stage. All the following stages are labelled with a level 0. Exceptions: pathways with a transfer to another hospital, stays with palliative care, death of the patient during the stay, and requests for home hospitalisation or rehabilitation. The following data were used: date of stay, diagnoses related group, time reference for the diagnoses related group, discharge destination to determine whether the patient died or was transferred, stay coding to determine whether palliative care occurred.

Algorithm 4 Rule 2: Evaluation the length of stay.

Let *reference* be the national reference of length of stay that corresponds to the diagnoses related group.
if the patient is transferred to another hospital for a serious reason or presumed discharge date is filled or received palliative care or died during the stay **then**
  the rule 2 is not applied.
end if

Let t_1 be the start date of the stay.
Let t_n be the end date of the stay.
Let $\sigma =< e_{11}, e_{12}, ..., e_{n1}, e_{n2} >$ be the trace representing the history pathway.
if $\#_{duration}(\sigma) > 1.5 * reference$ **then**
 $t_{end} = t_1 + reference$
 if t_{end} is a Saturday or a Sunday and t_n >next Monday 20h **then**
  t_{end} = next Monday at noon
 end if
 for e_{x1} such as $e_{x1} = (mu, start, tx_1)$ with $t_{x1} < t_{end}$ and $e_{x2} = (mu, end, t_{x2})$ with $t_{x2} > t_{end}$ **do**
  $e'_{x1} = ((mu, level2), start, t_{x1})$
  $e'_{x2} = ((mu, level2), end, t_{end})$
  $e'_{y1} = ((mu, level0), start, t_{end})$
  $e'_{y2} = ((mu, level0), end, t_{x2})$
 end for
 for each e_{x1} such as $e_{x1} = (mu, start, tx_1)$ with $t_{x1} > t_{end}$ **do**
  $e'_{x1} = ((mu, level0), start, t_{x1})$
  $e'_{x2} = ((mu, level0), end, t_{x2})$
 end for
end if

The last exception is addressed by another rule. Indeed, when a request for home hospitalisation or rehabilitation is made, a presumed discharge date is entered.

Rule 3: If the discharge date is later than the presumed discharge date, then the stage that begins before the presumed discharge date and ends after discharge is split into two stages: one stage (activity, level 2) from the start of the initial stage to the presumed discharge date and one stage (activity, level 0) from the presumed discharge date to the end of the initial stage. All the following stages are labelled with a level 0.

The data needed are the requests for home hospitalisation and rehabilitation. Notably, if a presumed discharge date was entered for every stay, rule 2 would not have been necessary.

Algorithm 5 Rule 3: Detection of delayed discharges caused by rehabilitation or home hospitalisation requests.

Let t_{presum} be the presumed discharge date.
if $t_n > t_{presum} + 1day$ **then**
  for e_{x1} such as $e_{x1} = (mu, start, t_{x1})$ with $t_{x1} < t_{presum}$ *and* $e_{x2} = (mu, end, t_{x2})$ with $t_{x2} > t_{presum}$ **do**
    $e'_{x1} = ((mu, level2), start, t_{x1})$ $e'_{x2} = ((mu, level2), end, t_{presum})$ $e'_{y1} = ((mu, level0), start, t_{presum})$
    $e'_{y2} = ((mu, level0), end, t_{x2})$
  end for
  for each e_{x1} such as $e_{x1} = (mu, start, t_{x1})$ with $t_{x1} > t_{presum}$ **do**
    $e'_{x1} = ((mu, level0), start, t_{x1})$
    $e'_{x2} = ((mu, level0), end, t_{x2})$
  end for
end if

Rule 4: If the activity is different from that of medically responsible unit, then the stage is labelled with a level 1.

Algorithm 6 Rule 4: Detection of overflow bed.

Let *mu* be a medical unit (activity) of the pathway and *mr* the unit medically responsible.
for each e_{x1} and $e_{x2} \in \sigma$ such as $e_{x1} = (mu, start, tx_1)$ $e_{x2} = (mu, end, t_{x2})$ **do**
  if $mu \neq mr$ **then**
    $e'_{x1} = ((mu, level1), start, t_{x1})$
    $e'_{x2} = ((mu, level1), end, t_{x2})$
  end if
end for

Rule 5: If a polyvalent unit is followed by a specialised unit (except the intensive care unit and continuous care unit) within 7 and a half days, then the stage of the polyvalent unit is labelled level 1.

Algorithm 7 Rule 5: Detection of polyvalent unit used as a buffer.

---

for each e_{x1} and $e_{x2} \in \sigma$ such as $e_{x1} = (mu, start, t_{x1})$ and $e_{x2} = (mu, end, t_{x2})$; the two events of stage s_x **do**
  if *mu* is a polyvalent unit and is not the last medical unit of the pathway and is not followed by an intensive care unit and $\#_{duration}(s_x) > 7\frac{1}{2}$ days **then**
    $e'_{x1} = ((mu, level1), start, t_{x1})$
    $e'_{x2} = ((mu, level1), end, t_{x2})$
  end if
end for

Rule 6: If the mutation is labelled as "awaiting bed" then the observation unit stage is labelled irrelevant (level 1). Daily and weekly hospitalisations only concern scheduled admissions. When an unscheduled patient begins his pathway in a daily or weekly hospitalisation unit, generally it means that no bed was available in the full hospitalisation unit.

Algorithm 8 Rule 6: Detection of observation unit used as a buffer.

for each e_{x1} and $e_{x2} \in \sigma$ such as $e_{x1} = (mu, start, t_{x1})$ and $e_{x2} = (mu, end, t_{x2})$ with $mu = \#_{activity}(e_{x1}) = \#_{activity}(e_{x2})$ = observation unit **do**
  if the mutation is labelled "awaiting bed" **then**
    $e'_{x1} = ((mu, level1), start, t_{x1})$
    $e'_{x2} = ((mu, level1), end, t_{x2})$
  end if
end for

Rule 7: If a weekly or daily hospitalisation unit is followed by the full hospitalisation unit, then the stage is labelled level 1.

Algorithm 9 Rule 7: Detection of daily and weekly hospitalisation units used as a buffer.

for each e_{x1} and $e_{x2} \in \sigma$ such as $e_{x1} = (mu, start, t_{x1})$ $e_{x2} = (mu, end, t_{x2})$ **do**
  if *mu* is a weekly hospitalisation unit and is not the last medical unit and the next activity is the associated full hospitalisation unit **then**
    $e'_{x1} = ((mu, level1), start, t_{x1})$
    $e'_{x2} = ((mu, level1), end, t_{x2})$
  end if
end for

Algorithm 10 Pathway correction specific for our dataset.

Let $\sigma' =< e'_{11}, e'_{12}, ..., e'_{m1}, e'_{m2} >$ be the labelled trace that starts at t_0 and ends at t_m.
Let *r* be the rule applied at e'_x.
Let $\sigma'' =< e''_{11}, e''_{12}, ..., e''_{m1}, e''_{m2} >$ be a copy of σ'.
for each $e''_x \in \sigma$ **do**
  if $\#_{activity}(e''_x)$ = (mu, level1) and *r* =rule 1 or rule 5 or rule 6 **then**
    $\#_{activity}(e''_x)$ = the next relevant activity
  else if $\#_{activity}(e''_x)$ = (mu, level1) and *r* = rule 4 **then**
    $\#_{activity}(e''_x)$ = the activity medically responsible
  else if $\#_{activity}(e''_x)$ = (mu, level1) and *r* = rule 7 **then**
    $\#_{activity}(e''_x)$ = the full hospitalisation unit of the same speciality
  else if $\#_{activity}(e''_x)$ = (mu, level0) **then** e''_x is deleted from σ''
  else $\#_{activity}(e''_x)$ = the medical unit of $\#_{activity}(e'_x)$
  end if
end for

for each $e''_x \in \sigma''$ with x < m **do**
  if $\#_{activity}(e''_{x1}) = \#_{activity}(e''_{x+1,1})$ **then**
    $\#_{time}(e''_{x2}) = \#_{time}(e''_{x+1,2})$ with $\#_{trans}(e''_{x+1,2})$ = complete and all the events of stage *x+1* are deleted from σ''
  end if
end for
Return $\sigma'' =< e''_{11}, e''_{12}, ..., e''_{p1}, e''_{p2} >$, the corrected trace.

Table S1: Correction of the pathways

Rule	Correction
Rule 1	The irrelevant ED stage is replaced by the next relevant stage.
Rule 2 and 3	The irrelevant stages are deleted.
Rule 4	The real medical unit is replaced by the unit medically responsible.
Rule 5	The activity name polyvalent unit, is replaced by the activity name of the next relevant stage.
Rule 6	The activity name observation unit, is replaced by the activity name of the next relevant stage.
Rule 7	The activity name of the weekly or daily hospitalisation is replaced by the full hospitalisation unit of the same speciality.
