# Supplementary material for: Evaluating the Bias in Hospital Data: Automatic Preprocessing of Patient Pathways Algorithm Development and Validation Study
Source: JMIR Med Inform. 2024 Sep 23;12:e58978. doi: 10.2196/58978 (PMC11459108; doi:10.2196/58978)
Supplement: Multimedia Appendix 3 [file medinform_v12i1e58978_app3.pdf]

Appendix 3: Statistical Analysis Tables

Table S2: Bivariate Analysis for Categorical Features

		Proportion (%)		*P* value
	**Features**	**ED visit ≤ 5h**	**ED visit > 5h**	
ED visits	**Weekday**			< .001
	Monday	16	20	
	Tuesday	14	16	
	Wednesday	15	14	
	Thursday	15	13	
	Friday	16	14	
	Saturday	13	11	
	Sunday	11	12	
	**Season**			0.513
	Spring	26	25	
	Summer	25	25	
	Autumn	24	24	
	Winter	25	26	
	**Arrival Period**			< .001
	Morning	19	16	
	Afternoon	33	21	
	Night	34	33	
	Deep Night	14	29	
	**Next Stage is Irrelevant**			0.053
	True	14	13	
	False	86	87	
ED visits	**History Next Stage**			< .001
	3O Surgery	6	5	
	Cardiology	2	3	
	Cardiology ICU	2	1	
	CCU	1	1	
	Geriatric Medicine	9	13	
	ICU	1	1	
	Nephrology Endocrinology	1	1	
	Neurology	1	2	
	Neurology ICU	2	3	
	Observation Unit	22	13	
	Observation Unit Villeneuve	9	5	
	Oncology Haematology Hepatogastro-enterology	4	6	
	Orthopaedic Surgery	5	3	
	Polyvalent Medicine	14	17	

	Post-emergency Villeneuve	4	3	
	Post-emergency	8	11	
	Pulmonology	3	4	
	Rheumatology	2	3	
	Visceral Surgery	4	5	
ED visits	**Corrected Next Stage**			< .001
	3O Surgery	1	1	
	Cardiology	3	4	
	Cardiology ICU	2	1	
	CCU	1	1	
	Geriatric Medicine	11	15	
	ICU	1	1	
	Nephrology Endocrinology	1	1	
	Neurology	2	3	
	Neurology ICU	2	3	
	Observation Unit	20	12	
	Observation Unit Villeneuve	8	4	
	Oncology Haematology Hepatogastro-enterology	5	8	
	Orthopaedic Surgery	6	4	
	Polyvalent Medicine	15	17	
	Post-emergency Villeneuve	3	3	
	Post-emergency	7	9	
	Pulmonology	4	5	
	Rheumatology	3	4	
	Visceral Surgery	5	5	
		**ED visit ≤ 10h**	**ED visit > 10h**	
ED visits	**Weekday**			< .001
	Monday	16	25	
	Tuesday	14	19	
	Wednesday	15	13	
	Thursday	15	10	
	Friday	16	9	
	Saturday	13	8	
	Sunday	11	16	
	**Season**			< .001
	Spring	26	21	
	Summer	25	27	
	Autumn	24	24	
	Winter	25	28	
	**Arrival Period**			< .001
	Morning	20	1	
	Afternoon	32	7	
	Night	34	39	
	Deep Night	14	53	

	**Next Stage Irrelevant**			< .001
	True	14	10	
	False	86	90	
ED visits	**History Next Stage**			< .001
	3O Surgery	6	2	
	Cardiology	2	4	
	Cardiology ICU	2	0	
	CCU	1	1	
	Geriatric Medicine	9	20	
	ICU	1	1	
	Nephrology Endocrinology	1	1	
	Neurology	2	2	
	Neurology ICU	2	3	
	Observation Unit	21	9	
	Observation Unit Villeneuve	9	1	
	Oncology Haematology Hepatogastro-enterology	4	7	
	Orthopaedic Surgery	5	2	
	Polyvalent Medicine	14	21	
	Post-emergency Villeneuve	4	1	
	Post-emergency	8	14	
	Pulmonology	3	4	
	Rheumatology	2	3	
	Visceral Surgery	4	3	
ED visits	**Corrected Next Stage**			< .001
	3O Surgery	1	0	
	Cardiology	3	4	
	Cardiology ICU	2	0	
	CCU	1	1	
	Geriatric Medicine	11	20	
	ICU	1	1	
	Nephrology Endocrinology	1	2	
	Neurology	2	3	
	Neurology ICU	2	3	
	Observation Unit	20	8	
	Observation Unit Villeneuve	8	1	
	Oncology Haematology Hepatogastro-enterology	5	9	
	Orthopaedic Surgery	6	1	
	Polyvalent Medicine	15	20	
	Post-emergency Villeneuve	3	1	
	Post-emergency	7	12	
	Pulmonology	4	5	
	Rheumatology	3	3	

	Visceral Surgery	5	3	
		**Relevant stage**	**Overflow stage**	
Overflow Beds	**Weekday**			< .001
	Monday	15	15	
	Tuesday	16	16	
	Wednesday	15	15	
	Thursday	15	13	
	Friday	16	13	
	Saturday	13	14	
	Sunday	10	13	
	**Season**			0.782
	Spring	27	27	
	Summer	25	25	
	Autumn	23	22	
	Winter	25	26	
	**Arrival Period**			< .001
	Morning	10	6	
	Afternoon	37	27	
	Night	31	36	
	Deep Night	22	30	
		**Prompt Discharge**	**Delayed Discharge**	
Delayed Discharge	**Discharge Destination**			< .001
	Home	70	63	
	Nursing home or home hospitalisation	4	5	
	Rehabilitation centre	15	27	
	Psychiatry	1	2	
	Transfer	2	1	
	Death	7	2	
	**Last Stage**			< .001
	**Season**			< .001
	Spring	18	16	
	Summer	16	57	
	Autumn	33	13	
	Winter	34	15	

Table S3: Bivariate Analysis for Numerical Features

	Features	Mean (Median)	(Q1, Q3)	Mean (Median)	(Q1, Q3)	*P* Value
		**ED visit ≤ 5h**		**ED visit >5h**		
		n = 16784 (83 %)		n = 3512 (17 %)		
**ED Visits** [a]	Age	68.5 (73)	(56, 85)	70.7 (75)	(60, 85)	<.001
	ED crowds	25.2 (25)	(18, 32)	23.9 (24)	(16, 31)	<.001
	Occupation Rate History Next Stage	85.7 (90.6)	(73.55, 100)	91.0 (96)	(83, 104)	<.001
	Occupation Rate Corrected Next Stage	87.3 (92.8)	(78.4, 100)	92.1 (97.6)	(88, 104)	<.001
		**ED visit ≤ 10h**		**ED visit >10h**		
		n = 18888 (93 %)		n = 1408 (7 %)		
**ED Visits** [a]	Age	68.6 (73)	(56, 85)	72.6 (76)	(64, 86)	<.001
	Occupation Rate History Next Stage	86.0 (91.2)	(74, 100)	94.5 (100)	(91.2, 106)	<.001
	Occupation Rate Corrected Next Stage	87.6 (93)	(79.2, 100)	94.8 (100)	(92.8, 106)	<.001
**Overflow Beds**		**Overflow Phase**		**Relevant Phase**		
		n = 2800 (11.28 %)		n = 22014 (88.72 %)		
	Arrival Hour [b]	13.9 (16)	(10, 19)	13.8 (15)	(11, 18)	0.365
	Occupation Rate Corrected Unit [b]	87.0 (95.2)	(85.6, 100)	85.4 (90)	(75.2, 98.6)	<.001
	ED crowds [c]	n = 1971 (13.18 %)		n = 12979 (86.82 %)		
		25.5 (25)	(19, 32)	24.4 (25)	(17, 31)	<.001
	Age [d]	n = 538 (3.65 %)		n = 14205 (96.35 %)		
		65.3 (68)	(52, 83)	67.2 (71)	(54, 84)	0.033
		**Delayed Discharge**		**Prompt Discharge**		
		n = 3550 (25.32 %)		n = 10471 (74.68 %)		
**Delayed Discharge**	Age	70.5 (74)	(60, 85)	70.4 (74)	(60, 85)	0.722

a. The analysis was done only on the data of the principal site (Scorff) because ED crowds and age are very different between the principal site and the smaller one (Villeneuve).
b. We compared the set of relevant stages and irrelevant stages.
c. In each pathway, the ED crowds was only computed for the first medical unit subsequent to the ED stage.
d. We compared the set of pathways without overflow stage and the set of pathways with at least one overflow stage.
