# Supplementary material for: Evaluating the Bias in Hospital Data: Automatic Preprocessing of Patient Pathways Algorithm Development and Validation Study
Source: JMIR Med Inform. 2024 Sep 23;12:e58978. doi: 10.2196/58978 (PMC11459108; doi:10.2196/58978)
Supplement: Multimedia Appendix 4 [file medinform_v12i1e58978_app4.pdf]

Appendix 4: Process Models

Figures legend:
S-HC 3 O(ORL-OPH-ORT): ENT, Ophthalmology, Orthopaedic Surgery
S-HC CARDIOLOGIE: Cardiology
S-HC CHIR ORTHO: Orthopaedic Surgery
S-HC CHIR VISCERALE: Visceral surgery
S-HC HGE: Hepato-Gastro-Enterology
S-HC MED PO: Polyvalent Medicine
S-HC MGG: Geriatric Medicine
S-HC NEPHRO ENDOC: Nephrology Endocrinology
S-HC NEUROLOGIE: Neurology
S-HC ONCO-HEMATO: Oncology Haematology
S-HC PNEUMOLOGIE: Pulmonology
S-HC RHUMATO-MI: Rheumatology Infectious Diseases
S-HC UNV: Neuro-vascular
S-HJ CARDIO: Cardiology Daily Hospitalisation
S-MPU: Post-Emergency Care
S-SAU: Emergency Department
S-UHCD: Observation Unit
S UNITE SAISON URG: Seasonal Unit
S-URLO: ICU
S-USCO: CCU
S-USIC: Cardiology ICU
S-USINV: Neurology ICU

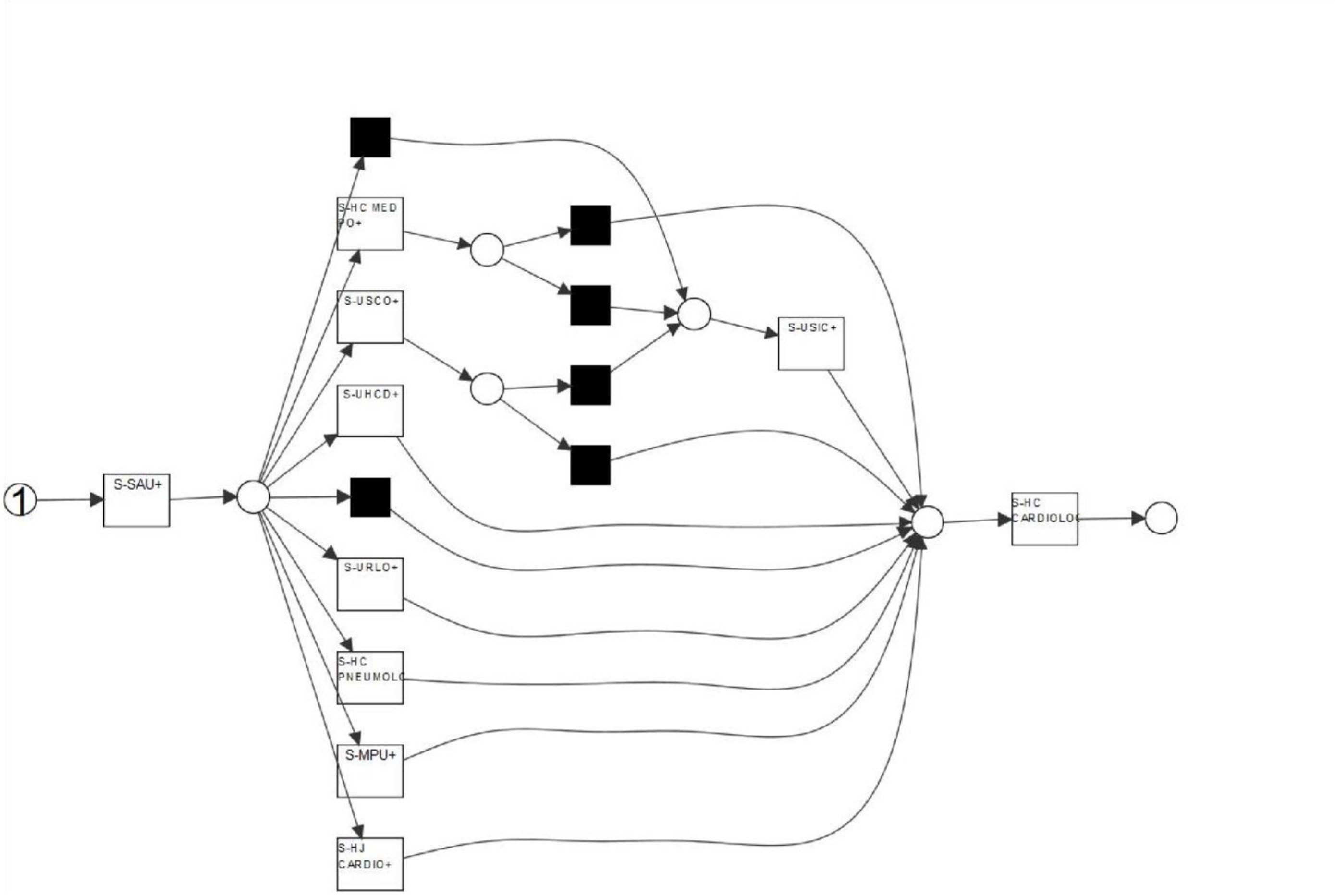

(a) History

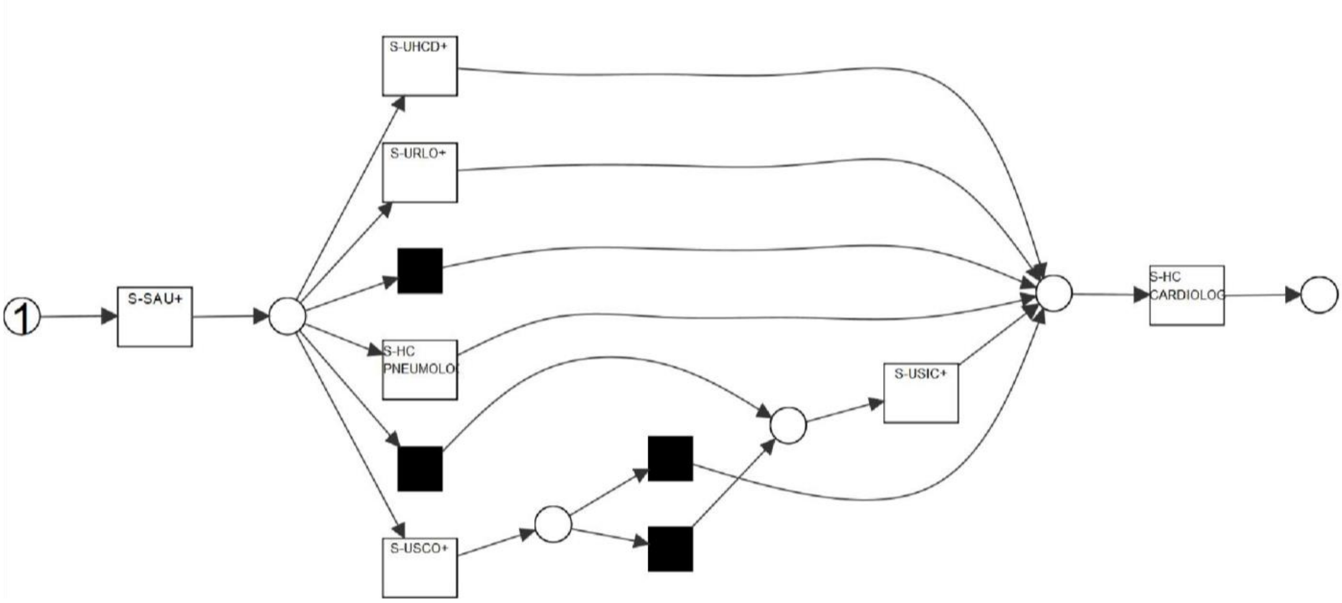

(b) Corrected

Figure S1: Cardiology graphs

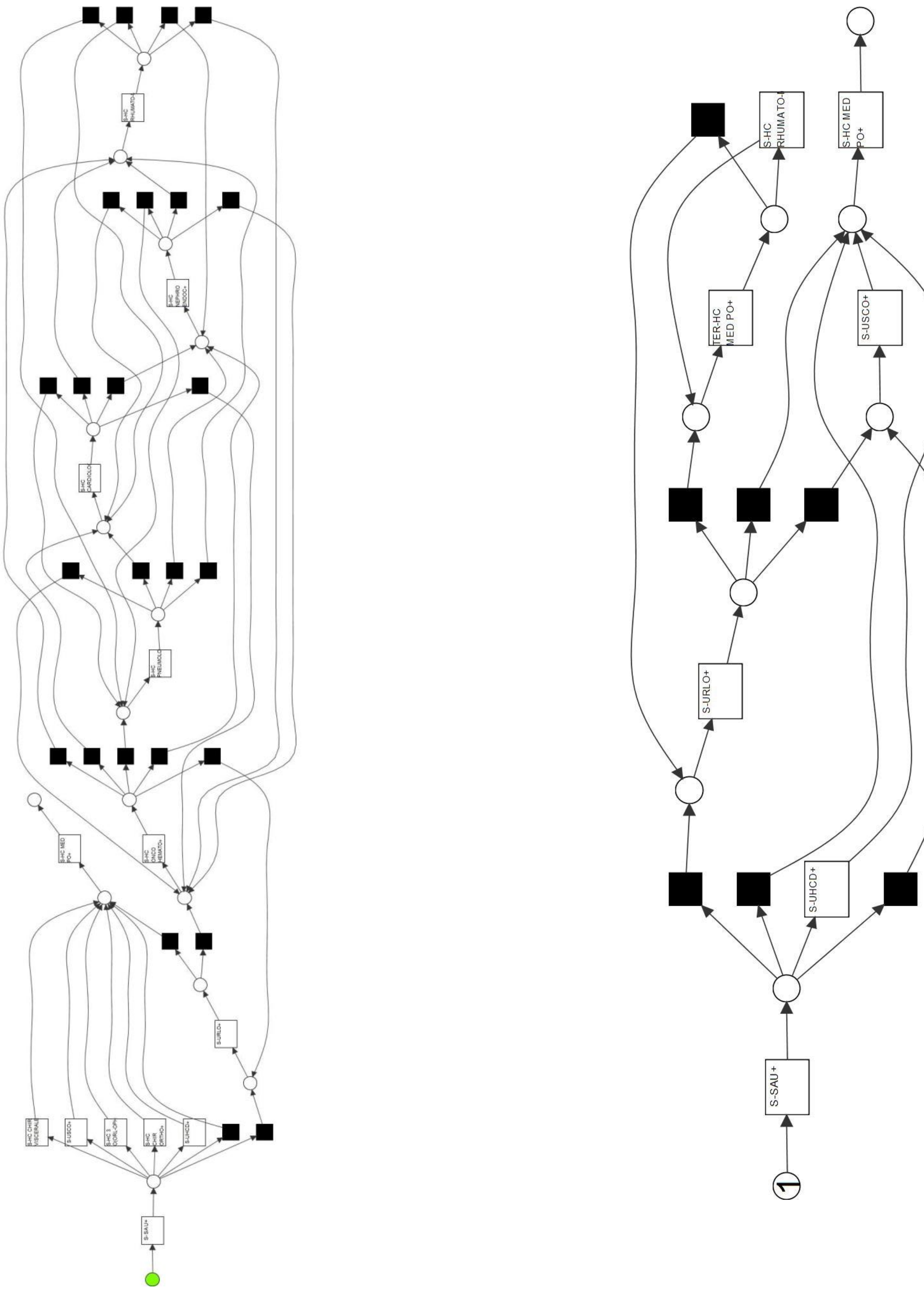

(a) History (b) Corrected

Figure S2: Polyvalent Medicine graphs

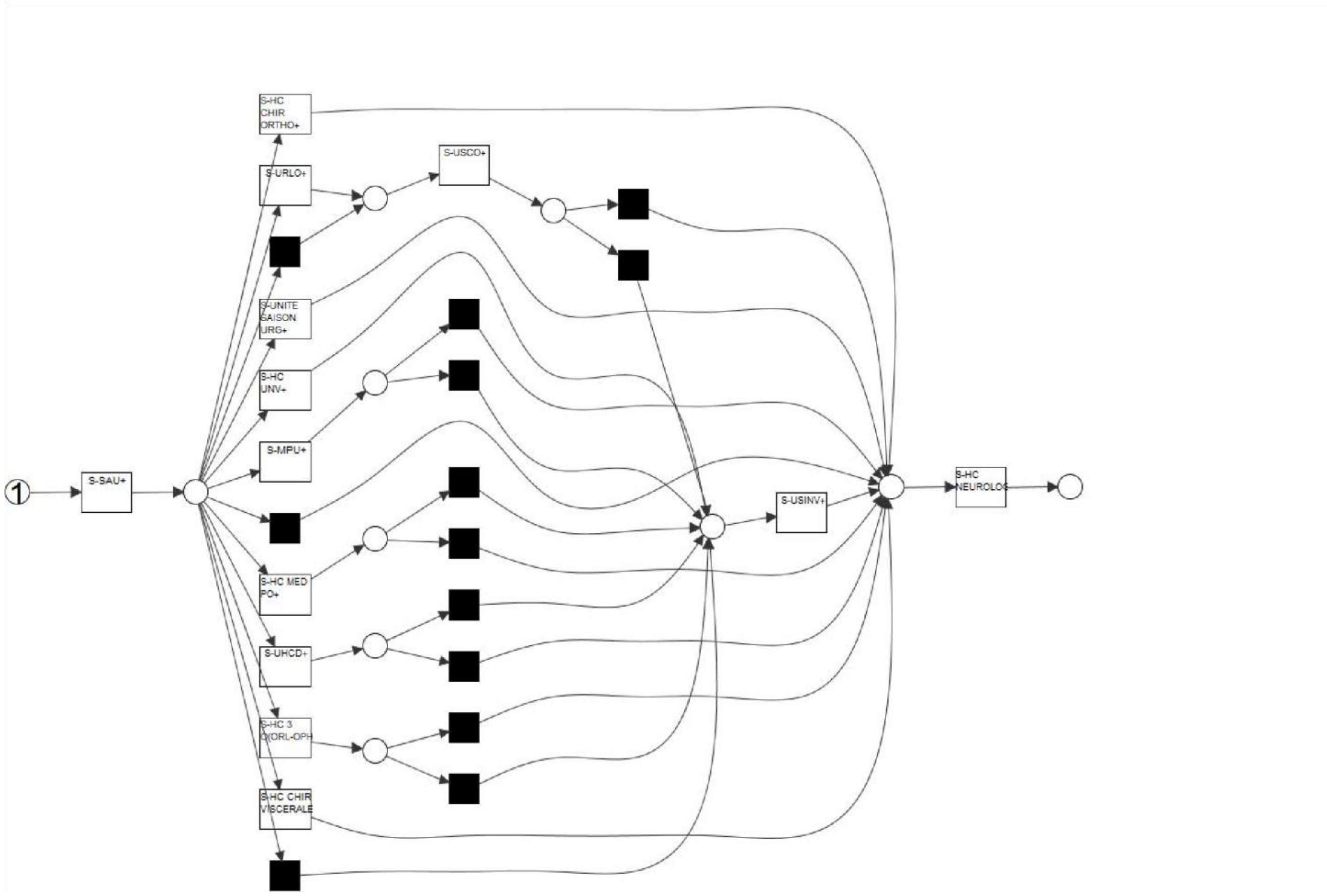

(a) History

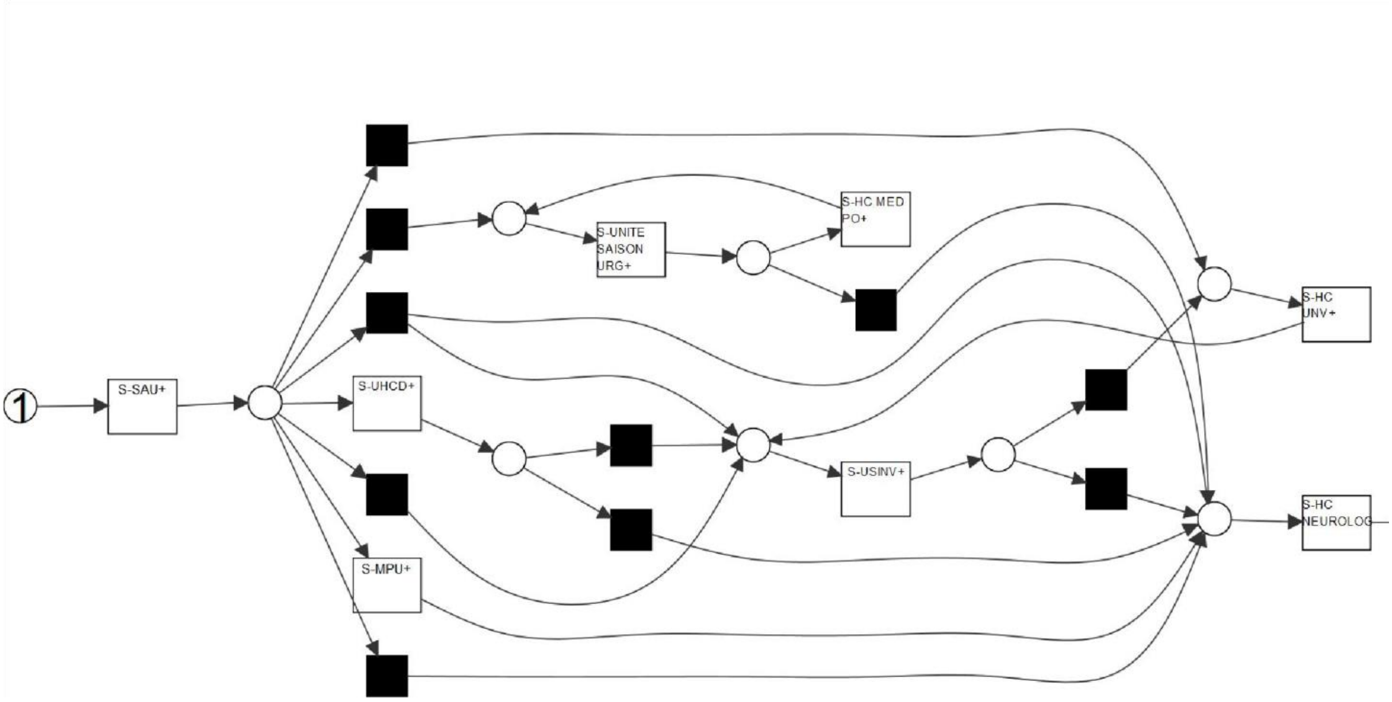

(b) Corrected

Figure S3: Neurology graphs

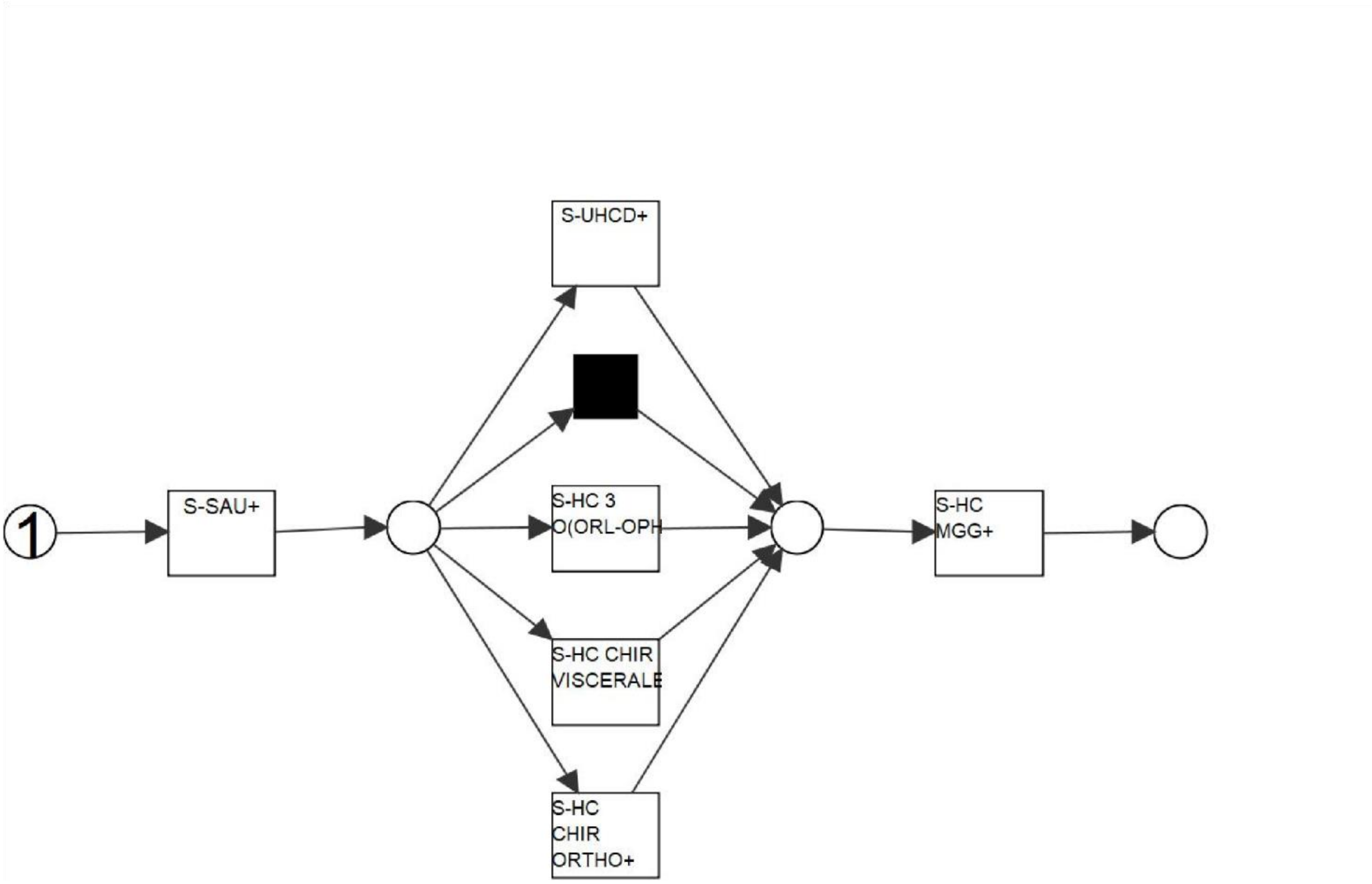

(a) History

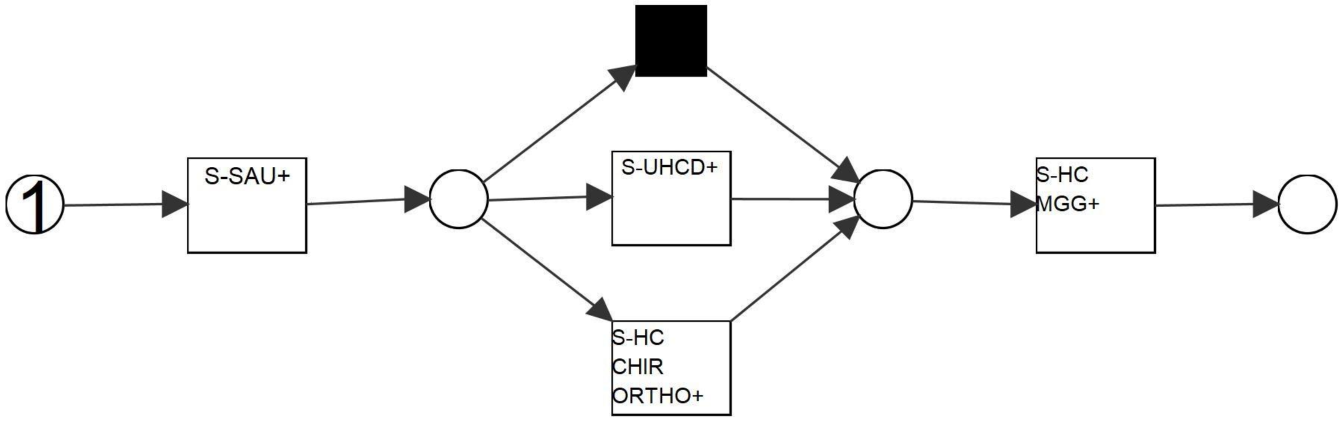

(b) Corrected

Figure S4: Geriatrics graphs

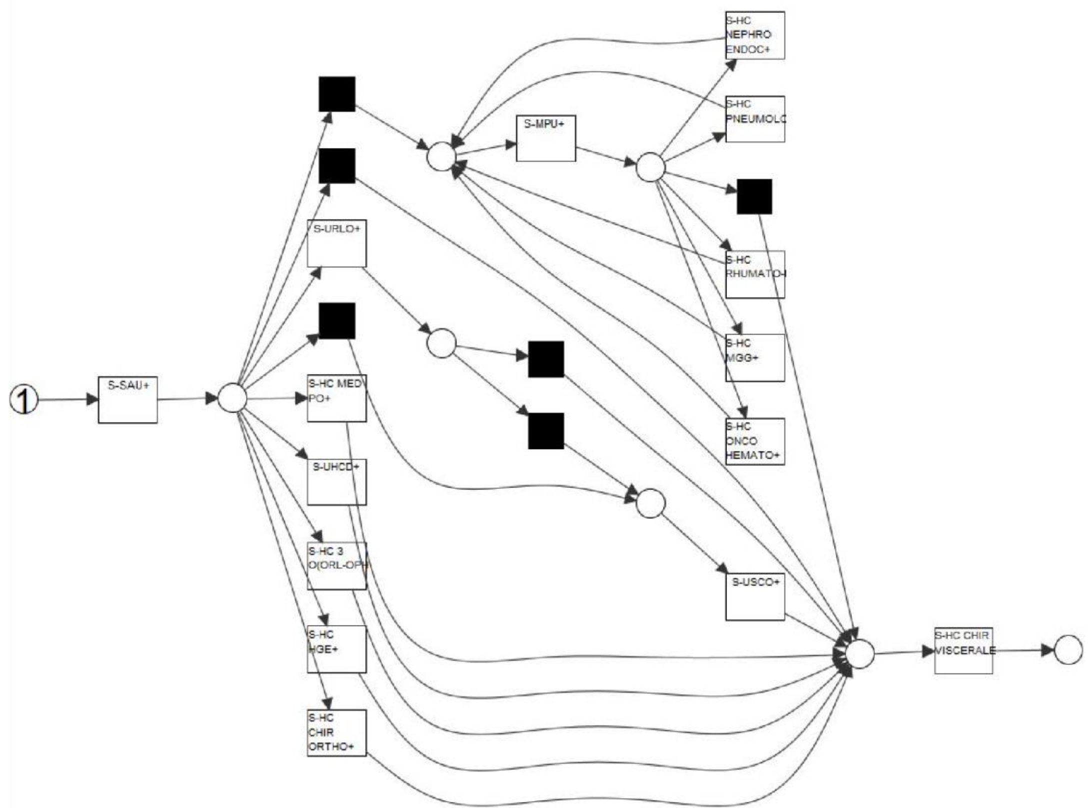

(a) History

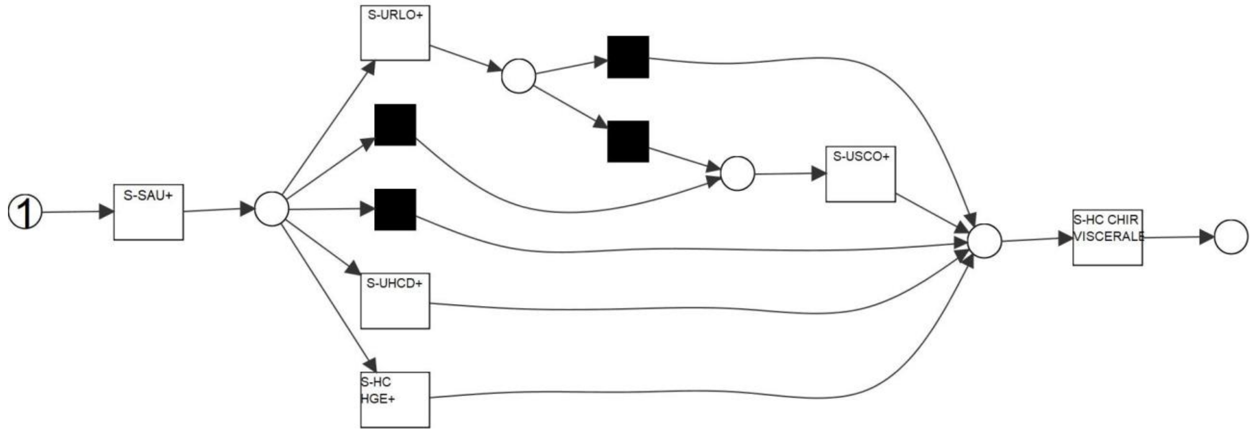

(b) Corrected

Figure S5: Visceral Surgery graphs
