# Supplementary material for: Evaluating the Bias in Hospital Data: Automatic Preprocessing of Patient Pathways Algorithm Development and Validation Study
Source: JMIR Med Inform. 2024 Sep 23;12:e58978. doi: 10.2196/58978 (PMC11459108; doi:10.2196/58978)
Supplement: Multimedia Appendix 5 [file medinform_v12i1e58978_app5.pdf]

Appendix 5: Simulation parametrisation

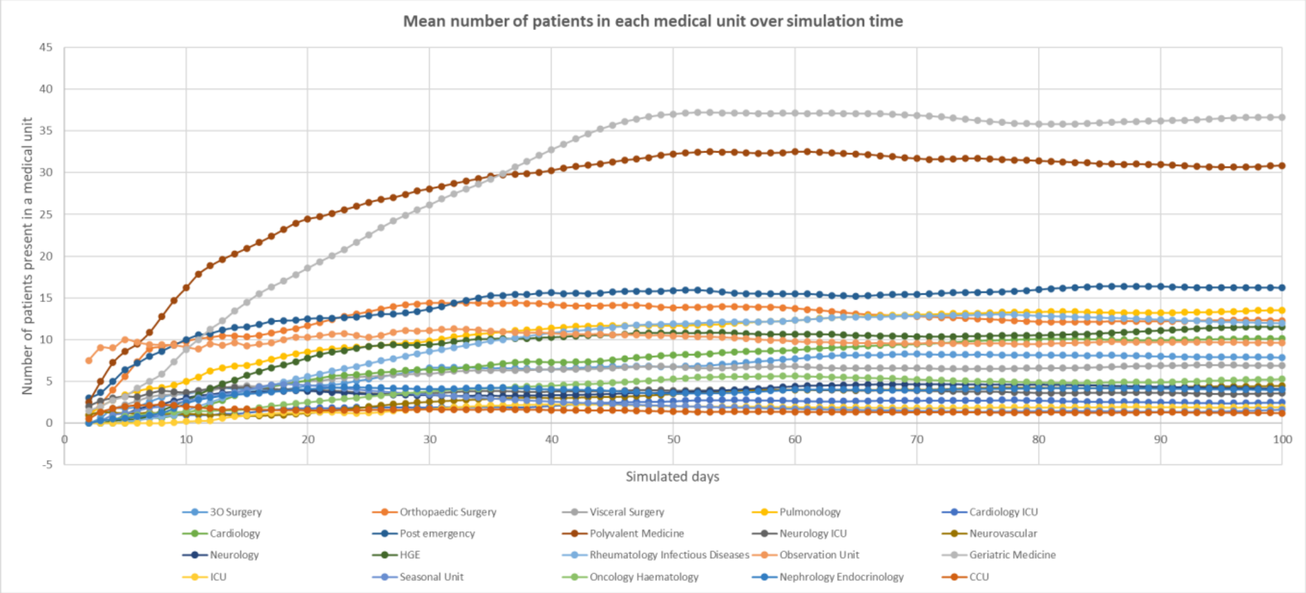

Figure S6: Mean number of patients in each medical unit over 100 simulated days

Table S4: Mean length of stay in days in each medical unit: from the real dataset and the simulation.

	**Simulation**		**Real dataset**		
**Medical unit**	**Mean**	**CI95**	**Mean**	**CI95**	**Absolute error**
3O Surgery	2.95	(2.75, 3.16)	3.06	(2.84, 3.28)	0.11
Cardiology	6.04	(5.57, 6.51)	5.72	(5.34, 6.1)	0.32
Orthopaedic Surgery	4.94	(4.57, 5.31)	5.04	(4.69, 5.4)	0.10
Visceral Surgery	3.49	(3.18, 3.79)	3.93	(3.59, 4.28)	0.45
Hepatogastroenterology	6.88	(6.44. 7,32)	6.98	(6.48, 7.48)	0.10
Polyvalent Medicine	10.00	(9.57, 10.42)	10.70	(10.13, 11.26)	0.70
Geriatric Medicine	13.50	(12.99, 14.02)	14.94	(14.1, 15.78)	1.44
Nephrology Endocrinology	8.25	(7.23, 9.26)	10.21	(8.87, 11.55)	1.96
Neurology	6.11	(5.15, 7.08)	7.59	(6.33, 8.85)	1.48
Oncology Haematology	11.71	(10.34, 13.07)	13.42	(11.26, 15.57)	1.71

Pulmonology	9.60	(8.89, 10.31)	9.59	(8.91, 10.27)	0.01
Rheumatology - infectious diseases	9.05	(8.35, 9.76)	9.18	(8.36, 10.0)	0.13
Neurovascular	6.51	(5.59, 7.44)	8.57	(6.81, 10.33)	2.06
Post emergency	5.13	(4.85, 5.42)	4.60	(4.35, 4.84)	0.54
Emergency Department	0.29	(0.28, 0.29)	0.26	(0.25, 0.26)	0.03
Observation Unit	0.67	(0.66, 0.68)	0.67	(0.65, 0.68)	0.00
Seasonal Unit	7.12	(5.94, 8.31)	6.51	(5.25, 7.77)	0.61
ICU	5.49	(4.48, 6.49)	4.98	(3.96, 6.0)	0.50
CCU	2.24	(1.95, 2.54)	2.43	(2.08, 2.78)	0.19
Cardiology ICU	3.05	(2.78, 3.32)	2.91	(2.7, 3.11)	0.14
Neurology ICU	3.54	(3.35, 3.74)	3.37	(3.18, 3.56)	0.17
Mean absolute error					0.61
